# New data from the Middle Jurassic of China shed light on the phylogeny and origin of the proboscis in the Mesopsychidae (Insecta: Mecoptera)

**DOI:** 10.1186/s12862-015-0575-y

**Published:** 2016-01-04

**Authors:** Xiaodan Lin, Matthew J. H. Shih, Conrad C. Labandeira, Dong Ren

**Affiliations:** College of Life Sciences, Capital Normal University, Beijing, China; Union County Magnet High School, Scotch Plains, NJ 07076 USA; Department of Paleobiology, National Museum of Natural History, Smithsonian Institution, Washington, DC, 20013 USA; Department of Entomology and BEES Program, University of Maryland, College Park, MD 20742 USA

**Keywords:** Evolutionary developmental model, Fossil insects, Inner Mongolia, Mesozoic, Morphological characteristics, Scorpionfly

## Abstract

**Background:**

The Mesopsychidae is an extinct family of Mecoptera, comprising eleven described genera from Upper Permian to Lower Cretaceous deposits. In 2009, several well-preserved mesopsychids with long proboscides were reported from the mid Mesozoic of Northeastern China, suggesting the presence of pollination mutualisms with gymnosperm plants and highlighting their elevated genus-level diversity. Since that time, additional mesopsychid taxa have been described. However, the phylogeny of genera within Mesopsychidae has not been studied formally, attributable to the limited number of well-preserved fossils.

**Results:**

Here, we describe two new species, *Lichnomesopsyche prochorista* sp. nov. and *Vitimopsyche pristina* sp. nov. and revise the diagnosis of *Lichnomesopsyche daohugouensis* Ren, Labandeira and Shih, 2010, based on ten specimens from the latest Middle Jurassic Jiulongshan Formation of Inner Mongolia, China. After compiling data from these new fossil species and previously reported representative taxa, we conducted phylogenetic analyses and geometric morphometric studies that now shed light on the taxonomy and phylogeny of Mesopsychidae. We also evaluate the recurring origin of the siphonate proboscis in the Mecoptera and propose an evolutionary developmental model for its multiple origins.

**Conclusions:**

Phylogenetic and geometric morphometric results confirm the establishment of two new species, each to *Lichnomesopsyche* and *Vitimopsyche. Vitimopsyche pristina* sp. nov. extends the existence of the genus *Vitimopsyche* Novokshonov and Sukacheva, 2001, from the mid Lower Cretaceous to the latest Middle Jurassic. Two methods of analyses indicate an affiliation of *Mesopsyche dobrokhotovae* Novokshonov, 1997 with *Permopsyc*he Bashkuev, 2011. A phylogenetic analysis of the Mesopsychidae supports: 1), Mesopsychidae as a monophyletic group; 2), *Mesopsyche* as a paraphyletic group, to be revised pending future examination of additional material; and 3), the independent origin of the proboscis in the Pseudopolycentropodidae, its subsequent loss in earliest Mesopsychidae such as *Epicharmesopsyche*, its re-origination in the common ancestor (or perhaps independently) in the *Vitimopsyche* and *Lichnomesopsyche* clades of the Mesopsychidae. The third conclusion indicates that the proboscis originated four or five times within early Mecoptera, whose origin is explained by an evolutionary developmental model.

**Electronic supplementary material:**

The online version of this article (doi:10.1186/s12862-015-0575-y) contains supplementary material, which is available to authorized users.

## Background

One of the iconic narratives in evolutionary biology is that associations between insects such as beetles, moths, flies and bees and flowering plants have resulted in an intricate nexus of coevolution between diversifying angiosperms and their insect pollinator clades from the mid Cretaceous to the present [1, 2]. In 2009, Ren et al. reported eleven species in Mesopsychidae, Aneuretopsychidae and Pseudopolycentropodidae with siphonate proboscides. The authors suggested that these mecopteran siphonate proboscides were used to feed on liquid pollination drops of gymnospermous reproductive structures and likely engaged in pollination mutualisms with gymnosperms during the mid-Mesozoic, long before the similar and independent establishment of coevolutionary associations between nectar-feeding bees, flies, moths, and beetles on angiosperms [3]. Recently, Peñalver et al. described two zhangsolvid flies from Late Cretaceous amber in Spain at 105 Ma (mega-annum) and Myanmar at 100 Ma that possess long proboscides. The Spanish specimen retained gymnosperm pollen adhering to the surface of its abdomen, thus providing evidence that these insects were engaged in pollination mutualisms with gymnosperms [4].

Mecoptera is a small order of insects comprising nine extant families [5] that contrasts with their greater diversity during the Mesozoic, encompassing about 39 extinct families and 207 genera described to date [6]. Within Mecoptera, Mesopsychidae Tillyard, 1917, are a small extinct family, and together with three other families, Aneuretopsychidae [7], Pseudopolycentropodidae [8], and Nedubroviidae [9], form a major clade of basal Mecoptera, the Aneuretopsychina [10], which have elongate, siphonate proboscides, feeding on pollination drops of gymnosperms [3, 11, 12]. Phylogenetic relationships among Mesopsychidae and other mecopteran-related families, including extinct basal panorpoids, extinct basal mecopterans and extant mecopterans previously have been reported [3, 13]. Currently, eleven genera with 27 species from the Upper Permian to the mid Lower Cretaceous have been assigned to Mesopsychidae [3, 14–24], which are summarized in Table 1. Fossil mesopsychids are known from Australia, Kyrgyzstan, South Africa, Ukraine, Tajikistan, Russia and China.

**Table 1:** Summary of all described genera and species of Mesopsychidae

Genus	Species	Geological age and locality	Preservation status	Reference
*Permopsyche*	*P. issadensis*	Upper Permian, Vologda Province, Russia.	Fore- and hind wings.	Bashkuev, 2011 [22]
	*P. rasnitsyni*	Upper Permian, Vologda Province, Russia.	Fore- and hind wings.	Bashkuev, 2011 [22]
	*P. robustus*	Upper Permian, Queensland, Australia.	Incomplete forewing only.	Riek, 1953 [16]
	*P. belmontensis*	Upper Permian, Queensland, Australia.	Fore- and hind wings.	Riek, 1953 [16]
*Mesopsyche*	*M. incompleta*	Uppermost Permian, Vologda Province, European Russia.	Incomplete fore- and hind wings.	Bashkuev, 2011 [22]
	*M. javorskii*	Uppermost Permian or Lower Triassic, Vladimir Province, Russia.	Hind wing only.	Zalessky, 1935 [54]
	*M. tongchuanensis*	Middle Triassic, Shaanxi Province, China.	Forewing only.	Hong, 2007 [20]
	*M. shcherbakovi*	Middle–Upper Triassic, Osh Region, Kyrgyzstan.	Fore- and hind wings.	Novokshonov, 1997 [18]
	*M. ordinate*	Middle–Upper Triassic, Osh Region, Kyrgyzstan.	Fore- and hind wings.	Novokshonov and Sukatsheva, 2001 [19]
	*M. justa*	Middle–Upper Triassic, Osh Region, Kyrgyzstan.	Fore- and hind wings.	Novokshonov and Sukatsheva, 2001 [19]
	*M. tortiva*	Middle–Upper Triassic, Osh Region, Kyrgyzstan.	Fore- and hind wings.	Novokshonov and Sukatsheva, 2001 [19]
	*M. gentica*	Middle–Upper Triassic, Osh Region, Kyrgyzstan.	Fore- and hind wings.	Novokshonov and Sukatsheva, 2001 [19]
	*M. triareolata*	Upper Triassic, Queensland, Australia.	Fore- and hind wings.	Tillyard, 1917 [14]
	*M. dobrokhotovae*	Upper Triassic, Khar’kov Region, Ukraine.	Fore- and hind wings.	Novokshonov, 1997 [18]
*Mesopanorpodes*	*M. wianamattensis*	Middle Triassic, New South Wales, Australia.	Incomplete forewing only.	Tillyard, 1918 [15]
*Mesoses*	*M. optata*	Upper Triassic, Bird’s River, South Africa.	Incomplete forewing only.	Riek, 1976 [17]
	*M. magna*	Upper Triassic, Bird’s River, South Africa.	Incomplete forewing only.	Riek, 1976 [17]
	*M. gayndah*	Early Middle Triassic, Gayndah, Australia.	Incomplete forewing only.	Lambkin, 2014 [24]
*Ptychopteropsis*	*P. mirabilis*	Lower Jurassic, Shurab, Tajikistan.	Forewing only.	Martynov, 1937 [55]
*Turanopsyche*	*T. venosa*	Lower Jurassic, Shurab, Tajikistan.	Hind wing only.	Martynov, 1937 [55]
*Ferghanopsyche*	*F. rotundata*	Lower Jurassic, Shurab, Tajikistan.	Forewing only.	Martynov, 1937 [55]
*Lichnomesopsyche*	*L. gloriae,*	Middle Jurassic, Inner Mongolia, China.	Complete body with wings.	Ren, Labandeira and Shih, 2010 [21]
	*L. daohugouensis*	Middle Jurassic, Inner Mongolia, China.	Complete body with wings.	Ren, Labandeira and Shih, 2010 [21]
*Epicharmesopsyche*	*E. pentavenulosa*	Middle Jurassic, Inner Mongolia, China.	Complete body with wings.	Shih, Qiao, Labandeira and Ren, 2013 [23]
*Vitimopsyche*	*V. torta*	Lower Cretaceous, Transbaikalia, Russia.	Forewing only.	Novokshonov and Sukacheva, 2001 [19]
	*V. kozlovi*	Lower Cretaceous, Hebei, China.	Complete body with wings.	Ren, Labandeira and Shih, 2010 [21]
*Baissopsyche*	*B. pura*	Lower Cretaceous, Transbaikalia, Russia.	Fore- and hindwings.	Novokshonov and Sukacheva, 2001 19]

Recently, we collected ten new specimens of mesopsychids from the latest Middle Jurassic Jiulongshan Formation at Daohugou Village, Shantou Township, from Ningcheng County of Inner Mongolia in Northeastern China. Based on these occurrences, two new species – *Lichnomesopsyche prochorista* sp. nov. and *Vitimopsyche pristina* sp. nov. – are described. In addition, the diagnosis of *Lichnomesopsyche daohugouensis* Ren, Labandeira and Shih, 2010, is revised based on five, well-preserved, new specimens of *L. daohugouensis* that display additional species-diagnostic characters of the hind wings which were lacking in the holotype [21].

*Vitimopsyche torta* was reported by Novokshonov in 2001 from the Lower Cretaceous Zaza Formation at Baissa, in Transbaikalian Russia [19]. Currently, two species, *V. torta* and *V. kozlovi*, have been assigned to *Vitimopsyche*, and both were described from the Lower Cretaceous. In this report, we describe a new species of *Vitimopsyche* from latest Middle Jurassic strata. This new find suggests that this genus has existed at least from the latest Middle Jurassic to the mid Lower Cretaceous and has had minimal morphological change for 40 million years [25–27].

Based on data from previously described, representative species and two new species erected herein, a suite of unique characters were chosen for phylogenetic analyses to shed light on the taxonomy, classification and phylogeny of the genera within Mesopsychidae. In addition, geometric morphometric analyses [28–31] for specimens with well-preserved forewings were conducted to supplement the phylogenetic analyses. Geometric morphometric analyses have been used in the study of phenetic relationships (without consideration of evolutionary relationships) among extant and fossil insects and their associations with cycadophyte, ginkgophyte and other land plants [28, 31–35]. The results from these two methods provide a preliminary understanding of the phylogenetic and phenetic relationships of selected genera of Mesopsychidae.

## Methods

### Examined taxa and terminology

The specimens described herein are housed in the fossil insect collection of the Key Laboratory of Insect Evolution and Environmental Changes, College of Life Sciences, Capital Normal University, Beijing, China (CNUB; Dong Ren, Curator). The specimens were examined using a Leica MZ 16.5 dissecting microscope connected to a Leica DFC500 digital camera, and illustrated with the aid of a drawing tube attached to the microscope. Photographs of entire specimens were taken with a Nikon D7000 digital camera coupled to a Nikkor 65 mm macro lens. The overlay drawings were prepared by using Adobe Illustrator CS6 and Adobe Photoshop CS5 graphics software. The wing venational nomenclature is based on Novokshonov (1997, 2002) [18, 36].

### Measurements and corresponding abbreviations

Body lengths were measured from the apex of the head, excluding antennae and proboscis, to the apex of the abdomen, excluding appendages. The wing lengths were measured from the base to the apex of wings. The lengths of the antennae were measured from their base to apex. Corresponding text and figure abbreviations are: Sc, subcosta; R1, first branch of the radius; Rs, radial sector; MA, anterior media; MP, posterior media; CuA, anterior cubitus; CuP, posterior cubitus; 1A, first branch of the anal vein; 2A, second branch of the anal vein.

### Phylogenetic analysis

We conducted phylogenetic analyses to elucidate the taxonomic and phylogenetic positions of the two new species and clarify the genus-level relationships of Mesopsychidae. Because many reported fossils lack well-preserved features of the body, only two body characters (characters 24 and 25) were used in this study (Table 2). The other 24 are wing characters; characters 0–22 are forewing characters (Table 2, Additional file 1: Figure S1), and character 23 is a hind wing character. The character selection partly is attributable to the characters used by Ren et al., 2009 [3] in their phylogenetic analysis of the Aneuretopsychina, or those scorpionflies characterized by siphonate, proboscate mouthparts. Thirteen mesopsychid species representing five genera with almost completely preserved forewing characters were selected as ingroups for the analysis (Tables 3 and 4). Described species with missing or poorly-preserved characters or possessing unusually inconsistent characters were excluded but summarized in Tables 3 and 4, with reasons for exclusion. *Mesopsyche* is represented by *M. triareolata, M. shcherbakovi* and *M. dobrokhotae,* but seven other species were excluded. *Mesopanorpodes wianamattensis* Tillyard, 1918 and *Baissopsyche pura* Novokshonov and Sukacheva, 2001 (the latter the only species in this genus) were also excluded. Three fossil species, *Permopanorpa inaequalis* Tillyard, 1926 (Permopanorpidae) [37, 38], *Protopanorpa longicubitalis* Bashkuev, 2010 (Permochoristidae) [39], and *Pseudopolycentropus janeannae* Ren, Labandeira and Shih, 2010 (Pseudopolycentropodidae) [8] were selected as outgroups for the analysis (Tables 3 and 4). The outgroup selection was based on the phylogeny of basal panorpids in Grimaldi and Engel, 2005 [13], and the phylogeny of Aneuretopsychina [3] showing their relation to basal panorpids and extant mecopterans. We chose the type genus, *Permopanorpa,* of Permopanorpidae as the root of the tree, because it is more basal than all other families in Aneuretopsychina. The character-state matrix, consisting of 16 taxa and 26 morphological characters with two or more character-states. This matrix is provided in Table 5.

**Table 2:** Definition of characters and their states

No.	Characters and their states
0	The relative level between Sc ending (Sc_2_) at C and CuA ending at posterior margin in the forewing: 0 – Sc ending far proximal to CuA ending; 1 – Sc ending at the same level with or slightly distal or proximal to CuA ending; 2 – Sc ending far distal from CuA ending.
1	The relative level of Sc fore-branch (Sc_1_) ending point vs. 1A ending point in the forewing: 0 – Sc_1_ ending point distal to 1A ending point; 1 – Sc_1_ terminus point very close or at the same level with 1A ending point; 2 – Sc_1_ ending point proximal to 1A terminus.
2	Presence of Sc_1_, branches of Sc_2_ and crossvein sc-r in forewing: 0 – with one Sc_2_ branch and one sc-r; 1 – with two Sc_2_ branches and one sc-r; 2 – no fore-branch (Sc_1_) and/or no sc-r.
3	The relative level between the point of MP origination from CuA and the point of RS + MA forking from R1 in the forewing: 0 – RS + MA forking from R1 slightly distal to MP forking from CuA; 1 – RS + MA forking from R1 far distal to MP forking from CuA.
4	The relative level between RS and MA forking points in the forewing: 0 – MA forking proximal to the RS forking; 1 – RS and MA forking points very close or at the same level; 2 – MA forking distal to the RS forking.
5	The relative level between MA and MP1 + 2 forking points in the forewing: 0 – MP1 + 2 (or MP1 + 2 + 3 + 4 in outgroup1 and outgroup 2) forking distal to the MA forking; 1 – MP1 + 2 forking very close to the MA forking; 2 – MP1 + 2 forking proximal to the MA forking.
6	Number of crossveins between R1 and C: 0 – no crossveins; 1 – 1 crossvein; 2 – 2 crossveins.
7	Presence of the crossvein r1-rs in the forewing: 0 – present; 1 – absent.
8	Number of RS branches in the forewing: 0 – 3 branches; 1 – 2 branches.
9	Number of MP branches in the forewing: 0 – >5 branches; 1 – 5 branches; 2 – 4 branches.
10	The relative level of MP forking point and RS + MA forking point in the forewing: 0 – MP forking proximal to the RS + MA forking; 1 – MP forking and RS + MA forking are very close or at the same level; 2 – MP forking distal to the RS + MA forking.
11	Posterior margin of forewings emarginated at the CuP vein apex: 0 –absent; 1 – present.
12	The inclination of the cup-cua (or cup-mp or CuA base) in the forewing: 0 – forming <90°angle with CuA; 1 – 90°or almost 90°angle with CuA; 2 – forming >90°angle with CuA.
13	The relative level of cup-cua (or cup-mp or CuA base) vs. MP origination from CuA in the forewing: 0 – cup-cua far distal to MP origination from CuA; 1 – cup-cua very close or slightly distal to MP origination from CuA; 2 – cup-cua proximal to MP origination from CuA.
14	The relative level of CuP and mid-length of the wing in the forewing: 0 – CuP proximal to mid-length of the wing; 1 – CuP almost at the same level or distal to mid-length of the wing.
15	The relative level of 2A ending vs. MP origination from CuA in the forewing: 0 –2A proximal to MP origination from CuA; 1 – 2A almost at the same level or distal to MP origination from CuA.
16	Symmetric spots on both wings: 0 – absent; 1 – present.
17	The relative level of crossvein rs2-ma (rs2-ma1 or rs-ma1) vs. MA1 + 2 forking point in the forewing: 0 – crossvein rs2-ma1 (rs-ma1) distal to MA1 + 2 forking point; 1 – crossvein rs2-ma proximal to MA1 + 2 forking point.
18	The relative level of crossvein rs2-ma (rs2-ma1 or rs-ma1 or rs3-ma1 for outgroup 2) vs. RS1 + 2 forking point in the forewing: 0 – crossvein rs2-ma (rs2-ma1 or rs3-ma1 for outgroup 2) distal to RS1 + 2 (RS1 + 2 + 3 for outgroup 2) forking point; 1 – crossvein rs-ma1 proximal to RS1 + 2 forking point.
19	The relative level of crossvein ma-mp1 + 2 vs. MA1 + 2 forking point in the forewing: 0 – crossvein ma-mp1 + 2 slightly distal to MA1 + 2 forking point; 1 – crossvein ma-mp1 + 2 slightly proximal to MA1 + 2 forking point; 2 – crossvein ma-mp1 + 2 far proximal to MA1 + 2 forking point.
20	The relative level of crossvein mp2-mp3 (mp1 + 2-mp3 or mp2 + 3-mp4) vs. MP1 + 2 (or MP 1 + 2 + 3) forking point in the forewing: 0 – crossvein mp1 + 2-mp3 proximal to MP1 + 2 forking point; 1 – crossvein mp2-mp3 slightly distal to MP1 + 2 forking point; 2 – crossvein mp2-mp3 much distal to MP1 + 2 forking point.
21	Number of crossveins (excluding sc-c, a1-a2 and a2-a3) in the forewing: 0 – > 10 crossveins; 1 – 8 – 10 crossveins; 2 – < 8 crossveins.
22	The anal area in the forewing: 0 –narrow; 1 – medium; 2 – expanded.
23	Length of Sc in the hind wing: 0 – short (proximal to MA bifurcation); 1 – medium (slightly proximal to MA bifurcation); 2 – long (distal to MA bifurcation).
24	Antennae: 0 – filiform and long antennae; 1 – short and filiform-like antennae; 2 – pectinate (comb-like) antennae.
25	Mouthparts: 0 – siphonate mouthparts not present; 1 – siphonate mouthparts present.

**Table 3:** Summary of the species included in the analyses

Included species	Phylogenetic analyses,	Geometric morphometric analyses		Comments
	Most parsimonious, tree, and Strict consensus tree	38 landmarks, w/o crossveins, Tree 1	42 landmarks, with two crossveins, Tree 2	
*Permopsyche rasnitsyni*	Yes	Yes	Yes	
*Permopsyche issadensis*	Yes	Yes	Yes	
*Permopsyche belmontensis*	Yes	Yes	Yes	
*Mesopsyche triareolata*	Yes	Yes	Yes	
*Mesopsyche dobrokhotovae*	Yes	Yes	Yes	
*Mesopsyche shcherbakovi*	Yes	Yes	Yes	
*Lichnomesopsyche gloriae*	Yes	Yes	Yes	
*Lichnomesopsyche daohugouensis*	Yes	Yes	Yes	
*Lichnomesopsyche prochorista*	Yes	Yes	Yes	
*Epicharmesopsyche pentavenulosa*	Yes	Yes	No	Most crossveins absent
*Vitimopsyche torta*	Yes	Yes	Yes	
*Vitimopsyche kozlovi*	Yes	Yes	Yes	
*Vitimopsyche pristina*	Yes	Yes	Yes	
*Permopanorpa inaequalis*	Yes	Yes	Yes	Outgroup 1
*Protopanorpa longicubitalis*	Yes	Yes	Yes	Outgroup 2
*Pseudopolycentropus janeannae*	Yes	Yes	Yes	Outgroup 3

The age and locality of three outgroups: (1) *Permopanorpa inaequalis*: Lower Permian, Noble County, Oklahoma, USA, Wellington Formation; (2) *Protopanorpa longicubitalis*: Lower Permian, Ufimian, Solikamsk Horizon, Tyulkino locality; (3) *Pseudopolycentropus janeannae*: Middle Jurassic, Inner Mongolia, China, Jiulongshan Formation

**Table 4:** Summary of the species excluded with reasons for exclusion

Excluded species	Reasons for exclusion
*Baissopsyche pura*	Forewing venation not complete, key characters missing
*Mesopsyche ordinata*	Forewing width/length ratio at 0.48 much higher than that of all others (with typical ratio between 0.30 and 0.35 and two outliers at 0.27 and 0.38)
*Mesopsyche justa*	Base of forewing missing for holotype; inconsistent venation among holotype and paratypes.
*Mesopsyche gentica*	Base of forewing missing
*Mesopsyche tortiva*	Incomplete forewing and base of hind wing missing
*Mesopsyche incompleta*	Incomplete fore- and hind wings
*Mesopsyche javorskii*	Hind wing only
*Mesopsyche tongchuanensis*	Incomplete forewing only
*Mesopanorpodes wianamattensis*	Anal area of forewing missing
*Permopsyche robustus*	Incomplete forewing only
*Mesoses optata*	Incomplete forewing only
*Mesoses magna*	Incomplete forewing only
*Mesoses gayndah*	Incomplete forewing only
*Ptychopteropsis mirabilis*	Incomplete forewing only
*Turanopsyche venosa*	Incomplete hind wing only
*Ferghanopsyche rotundata*	Incomplete forewing only

**Table 5:** Character state matrix of 26 characters for the 16 taxa included in the phylogenetic study

Taxa/ character	0										1										2					
	0	1	2	3	4	5	6	7	8	9	0	1	2	3	4	5	6	7	8	9	0	1	2	3	4	5
*Permopanorpa inaequalis* (Permopanorpidae)	1	0	0	0	0	0	2	0	0	0	0	0	0	0	0	0	0	0	0	0	1	0	1	0	?	?
*Protopanorpa longicubitalis*	2	0	0	0	1	0	2	0	0	0	0	0	0	1	0	1	0	0	0	1	0	0	1	?	?	?
(Permochoristidae)																										
*Pseudopolycentropus janeannae*	0	?	2	0	0	1	1	1	1	1	0	0	1	2	0	1	0	?	?	2	1	2	0	0	0	1
(Pseudopolycentropodidae)																										
*Permopsyche rasnitsyni*	1	0	0	1	2	2	0	0	1	2	0	1	1	1	1	1	0	0	0	2	1	1	2	?	?	?
*Permopsyche issadensis*	1	1	0	1	1	2	0	0	1	2	0	1	1	1	1	1	0	0	0	2	1	1	2	0	?	?
*Permopsyche belmontensis*	1	1	0	1	2	2	0	0	1	2	0	0	1	1	1	1	0	1	0	2	0	1	2	?	?	?
*Mesopsyche triareolata*	1	1	1	1	0	0	1	0	1	2	0	0	2	0	1	1	0	0	0	2	1	1	2	?	?	?
*Mesopsyche dobrokhotovae*	1	2	0	1	1	2	0	0	1	2	0	1	2	1	1	1	0	0	0	2	1	1	2	?	?	?
*Mesopsyche shcherbakovi*	2	0	0	1	1	2	0	0	1	2	0	1	1	1	0	0	0	0	0	1	1	1	2	?	?	?
*Lichnomesopsyche gloriae*	2	1	0	0	0	2	0	0	1	2	1	1	1	0	1	1	0	0	0	2	1	1	2	0	1	1
*Lichnomesopsyche daohugouensis*	2	1	0	0	1	2	0	0	1	2	1	1	1	0	1	1	1	0	0	2	1	1	2	0	1	1
*Lichnomesopsyche prochorista*	2	1	0	0	2	2	0	0	1	2	1	1	1	0	1	1	0	0	0	2	1	1	2	0	1	1
*Epicharmesopsyche pentavenulosa*	2	0	0	1	0	0	0	1	1	1	0	1	0	2	1	1	1	?	?	?	?	2	2	2	0	0
*Vitimopsyche pristina*	2	0	0	1	0	0	0	0	1	2	2	1	1	1	1	1	0	0	1	1	2	1	2	1	?	1
*Vitimopsyche torta*	2	0	1	1	0	0	0	0	1	2	2	?	1	1	1	1	0	0	0	1	2	1	2	?	?	?
*Vitimopsyche kozlovi*	2	0	0	1	0	0	0	0	1	2	2	1	1	1	1	?	0	0	1	1	?	1	2	0	2	1

The “?” denotes that the state is unknown or is inapplicable

The character-state matrix was entered into WinClada (Version1.00.08) [40], a morphological analysis software program. Tree searches implemented a heuristic search method, with options set to hold 10,000 trees, 1000 replications, 100 starting tree replication, and a multiple TBR + TBR search strategy. All characters were treated as unordered and were weighted equally. Missing or inapplicable characters were coded with question marks. Parsimony analyses were performed by using NONA (Version 2.0) [41] and PAUP (Version 4.0b10) [42]. We conducted both parsimony versions because only one outgroup can be defined in the NONA but three outgroups are identified in PAUP, resulting in different heuristic searches used in these two programs.

For NONA, we use an exhaustive search option and bootstrap support values from 1000 replications are presented as numbers under branch nodes. For PAUP, the same matrix editing program was used, Nexus Data Editor (Version 5.0), and the NEX file was entered into PAUP which employs a general heuristic search with 1000 replicates and TBR branch swapping. All characters were treated as unordered and equally weighted.

### Geometric morphometric analysis of forewings

We began our analyses by obtaining and compiling completed forewing samples from the literature, and then chose landmarks to identify their wing shape, relative positions of the branching points, and number of branches. The specimens, the same as those of the phylogenetic analyses, comprise 13 ingroups and three outgroups.

**Fig. 1:**
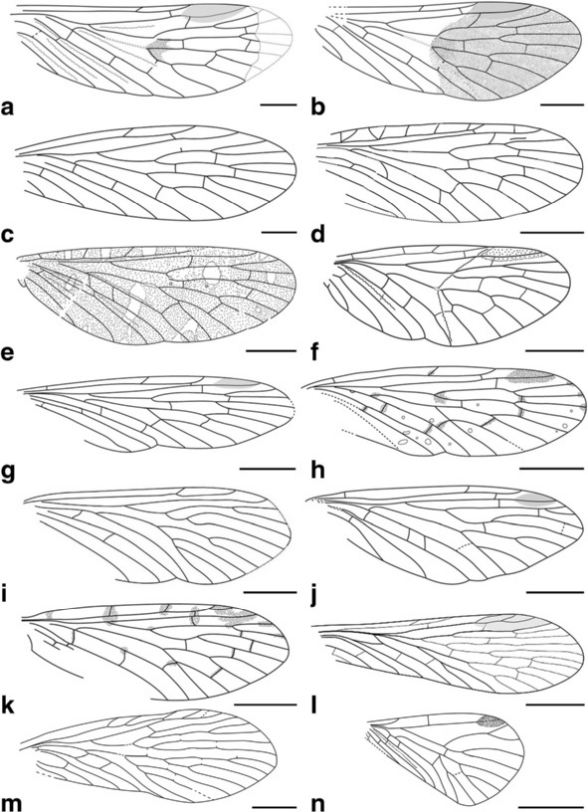
Line drawings of forewing samples for 14 representative species used in analyses. **a** *Permopsyche rasnitsyni*, from Bashkuev, 2011 [22]. **b** *Permopsyche issadensis*, from Bashkuev, 2011 [22]. **c** *Permopsyche belmontensis*, from Riek, 1953 [16] and Bashkuev, 2011 [22]. **d** *Mesopsyche triareolata*, from Bashkuev, 2011 [22] and Lambkin, 2014 [24]. **e** *Mesopsyche dobrokhotovae*, from Novokshonov, 1997 [18] and Bashkuev, 2011 [22]. **f** *Mesopsyche shcherbakovi*, from Novokshonov, 1997 [18]. **g** *Lichnomesopsyche gloriae*, from Ren, Labandeira, and Shih, 2010 [21]. **h** *Lichnomesopsyche daohugouensis*, from Ren, Labandeira, and Shih, 2010 [21]. **i** *Epicharmesopsyche pentavenulosa*, from Shih, Qiao, Labandeira, and Ren, 2013 [23]. **j** *Vitimopsyche kozlovi* from Ren, Labandeira, and Shih, 2010 [21] (one crossvein [between MP2 and MP3] estimated using other two species of the same genus). **k** *Vitimopsyche torta*, from Novokshonov and Sukacheva, 2001 [19] (two landmarks [for endings of 1A and 2A] estimated using average of the other two species of the genus). **l** *Permopanorpa inaequalis*, from Beckemeyer and Hall, 2007 [51]. **m** *Protopanorpa longicubitalis*, from Bashkuev, 2010 [39]. **n** *Pseudopolycentropus janeannae*, from Ren et al., 2010 [8]. Scale bars represent 5 mm in (**e**) and (**g**–**m**), 1 mm in (**a**–**c**), and 3 mm in (**d**), (**f**) and (**n**)

**Fig. 2:**
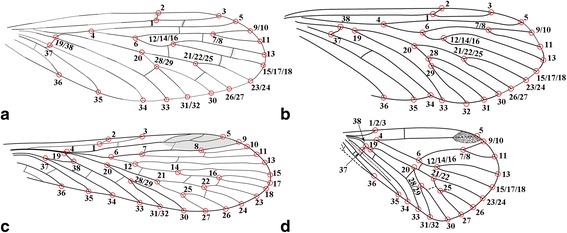
The landmark numbers of the four forewing samples for the case of 38 landmarks. **a** *Vitimopsyche pristina* sp. nov. (original). **b** *Epicharmesopsyche pentavenulosa*, from Shih, Qiao, Labandeira, and Ren, 2013 [23]. **c** *Permopanorpa inaequalis*, R. J. Tillyard, 1926 [37]. **d** *Pseudopolycentropus janeannae*, from Ren et al., 2010 [8]. Not to scale

We used Adobe Photoshop CS5 and Adobe Illustrator CS6 to re-draw the venation of the 13 ingroups and three outgroups based on line drawings published in the literature (Fig. 1), with minor modifications and assumptions noted in the Fig. 1 caption. The drawings were entered into tps-UTILS [43] to convert the JPEG files into TPS files for further use. For the first set of analysis, 38 landmarks were placed using tps-DIG [44] on positions of key veins on the wings, four examples of which are shown in Fig. 2a–d. Compared to the venation of *Vitimopsyche pristina* sp. nov. (Fig. 2a), the outgroup *Permopanorpa inaequalis* (Fig. 2c) has an extra RS, two extra MA veins, and two extra MP veins and *Protopanorpa longicubitalis* (Fig. 1m) has an extra RS and three extra MP veins, while the outgroup *Pseudopolycentropus janeannae* (Fig. 2d) and the ingroup *E. pentavenulosa* (Fig. 2b) have an extra MP vein*.* To accommodate these additional veins, we applied additional landmarks to each extra vein for representation (Fig. 2b–d). For all other wings that lack these extra veins, we simply doubled or occasionally tripled the number of landmarks as shown in Fig. 2a. A consensus was calculated by using all 16 samples and 38 landmarks in tps-SUPER [45]. Later, using the consensus as the “reference” and all sixteen samples as the “data”, we used tps-SPLIN [46] to calculate the Procrustes distances [47]. We converted the data into NTS files to complete the final step in NTedit where a matrix was produced and labeled. We entered this matrix into NTSYSpc [48], where we used an unweighted pair-group method using arithmetic averages (UPGMA) to produce the first known tree showing the phenetic relationships among these 16 scorpionfly wing samples based on 38 landmarks.

**Fig. 3:**
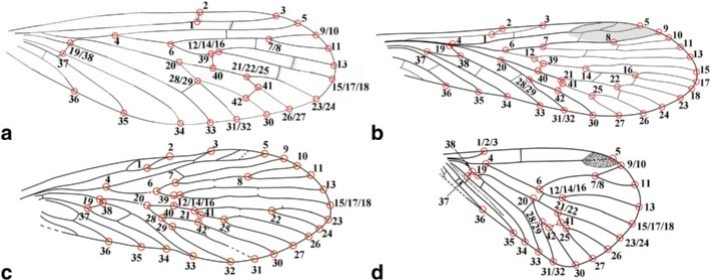
The landmark numbers of the four forewing samples for the case of 42 landmarks. **a** *Vitimopsyche pristina* sp. nov. (original). **b** *Permopanorpa inaequalis*, R. J. Tillyard (1926) [37]. **c** *Protopanorpa longicubitalis*, from Bashkuev (2010) [39]. **d** *Pseudopolycentropus janeannae*, from Ren et al. (2010) [8]. Not to scale

To conduct a more detailed analysis, we ran the above program again by adding positions of two crossveins with 42 landmarks (Fig. 3a–d). Because *E. pentavenulosa* does not have any of these crossveins, we decided to exclude this taxon from this set of analyses. The 15 images of 12 ingroups and three outgroups subsequently were entered into tps-UTILS, marked in tps-DIG, averaged in tps-SUPER, calculated in tps-SPLIN, and transferred to the second tree in NTSYSpc, using the same procedures mentioned above.

### Ethics statement

The authors declare that the study makes no uses of human, clinical tools and procedures, vertebrate and regulated invertebrate animal subjects and/or tissue, and plants.

## Results

### Systematic paleontology

Family Mesopsychidae Tillyard, 1917.

Type genus: *Mesopsyche* Tillyard, 1917.

*Lichnomesopsyche* Ren, Labandeira and Shih, 2010 [21].

Type species: *Lichnomesopsyche gloriae* Ren, Labandeira and Shih, 2010 [21].

Included species: Type species, *Lichnomesopsyche daohugouensis* Ren, Labandeira and Shih, 2010, and the new species described herein.

#### Emended diagnosis

In forewing, both Rs and MA with two endings, not always forking at the same level. The MP bifurcation is slightly proximal of Rs + MA bifurcation, or both forking at the same level. In the hind wing, MP originates from the stem of MP + CuA slightly distal or proximal of the Rs + MA originating from R1.

*Lichnomesopsyche prochorista* sp. nov.

urn: lsid:zoobank.org:act:0C5A060A-9314-4752-94FE-35B65B18A132

#### Diagnosis

The new species resembles *Lichnomesopsyche gloriae* and *L. daohugouensis* in venational features, but differs from both by Rs forking proximal of the MA bifurcation on the fore- and hind wings. It also differs from *L. daohugouensis* by not having spots on the fore- and hind wings.

#### Etymology

The specific name is a combination of Latin “*pro-*” (before) and the Greek “*choristos*” (separate), indicating that the forking of Rs vein is proximal of the MA vein bifurcation.

#### Holotype

**Fig. 4:**
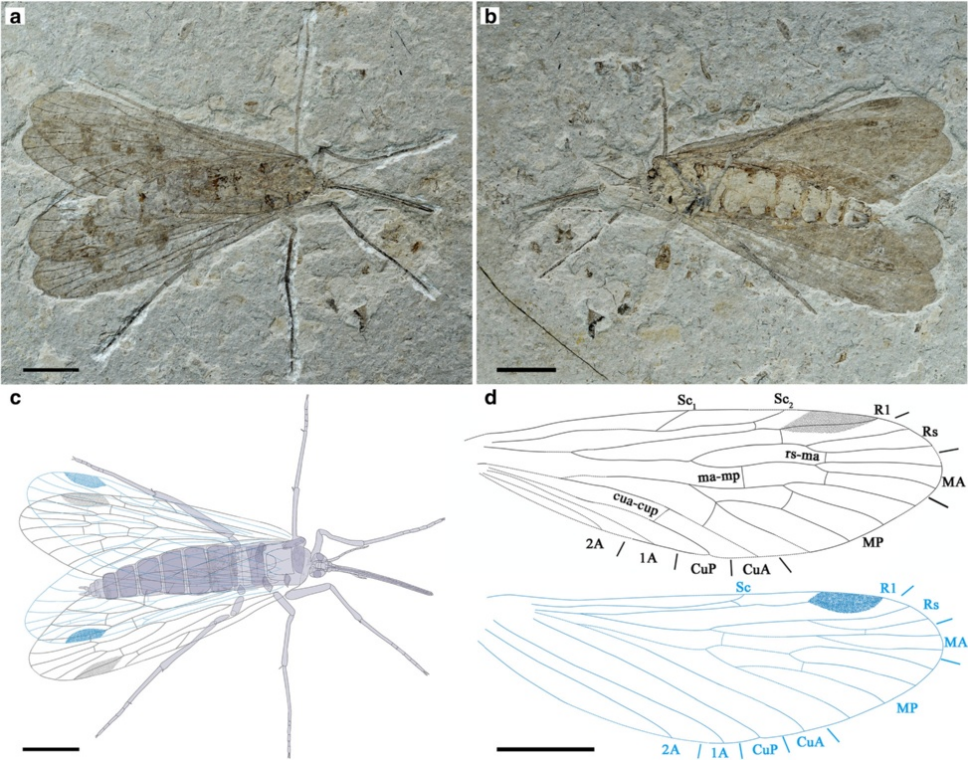
*Lichnomesopsyche prochorista* sp. nov., holotype specimen CNU-MEC-NN-2015002p/c. **a** Photograph of part. **b** Photograph of counterpart. **c** Overlay drawing of part. **d** Line drawings of right fore and hind wings. Scale bars represent 5 mm in (**a**–**d**)

**Fig. 5:**
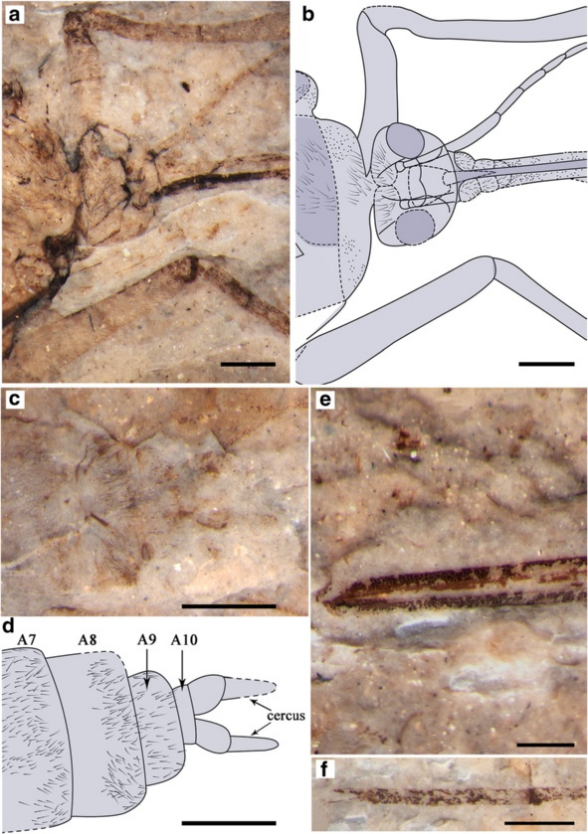
Detail structures of *Lichnomesopsyche prochorista* sp. nov., holotype specimen CNU-MEC-NN-2015002p. **a** Photograph under ethanol of head and antennae. **b** Overlay drawing of the head and antennae. **c** Photograph of genitalia with vestiture in dorsal view, under alcohol. **d** Overlay drawing of female genitalia in dorsal view. **e** Photograph under ethanol of the proboscis base. **f** Photograph under ethanol of tarsi and associated two claws of right foreleg. Scale bars represent 1 mm in (**a**–**d**) and (**f**), 0.5 mm in (**e**). Corresponding abbreviations are: A7: the seventh segment of the abdomen; A8: the eighth segment of the abdomen; A9: the ninth segment of abdomen; A10: the tenth segment of the abdomen

Specimen CNU-MEC-NN-2015002p/c, part and counterpart (Figs. 4 and 5) is an almost complete specimen with well-preserved body and wings; female. Forewing length 24.6 mm, width 7.5 mm; body length (excluding antennae and proboscis) 22.2 mm; proboscis length at least 9.7 mm; antenna length (as preserved) at least 4.8 mm.

#### Paratypes

**Fig. 6:**
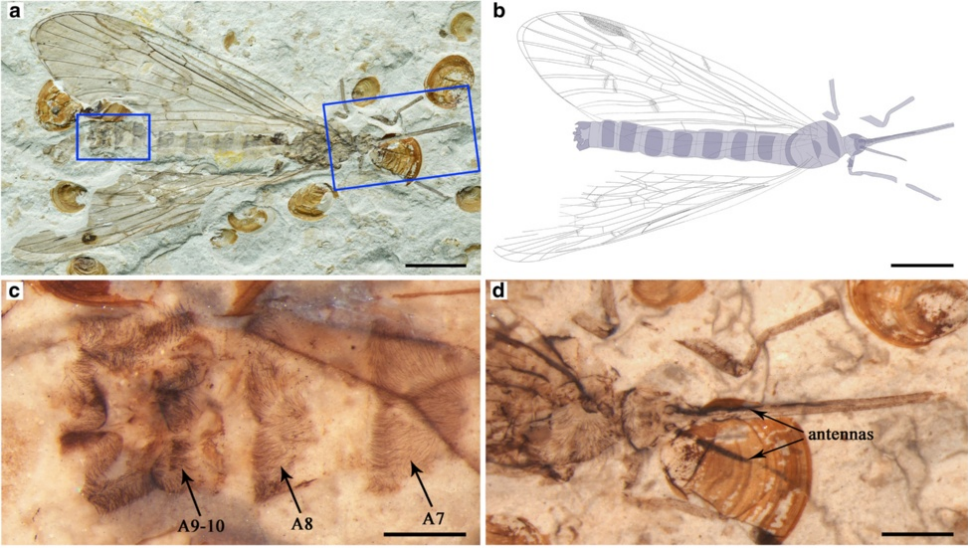
*Lichnomesopsyche prochorista* sp. nov., paratype specimen CNU-MEC-NN-2015008. **a** Photograph of specimen. **b** Overlay drawing of habitus. **c** Photograph under ethanol of male genitalia with vestiture in dorsal view. **d** Photograph under ethanol of head and part of thorax in dorsal view. Scale bars represent 5 mm in (**a**) and (**b**), 1 mm in (**c**), and 2 mm in (**d**). Corresponding abbreviations are: A7: the seventh segment of the abdomen; A8: the eighth segment of the abdomen; A9–10: the nine to tenth segments of the abdomen

Specimen CNU-MEC-NN-2015008, a well-preserved individual showing the complete insect in dorsal view (Fig. 6); male. Forewing length at least 25.9 mm, width about 7.7 mm; body length (as preserved, excluding antennae and proboscis) 23.5 mm; proboscis length (as preserved) at least 7.1 mm; antenna length at least 4.6 mm.

**Fig. 7:**
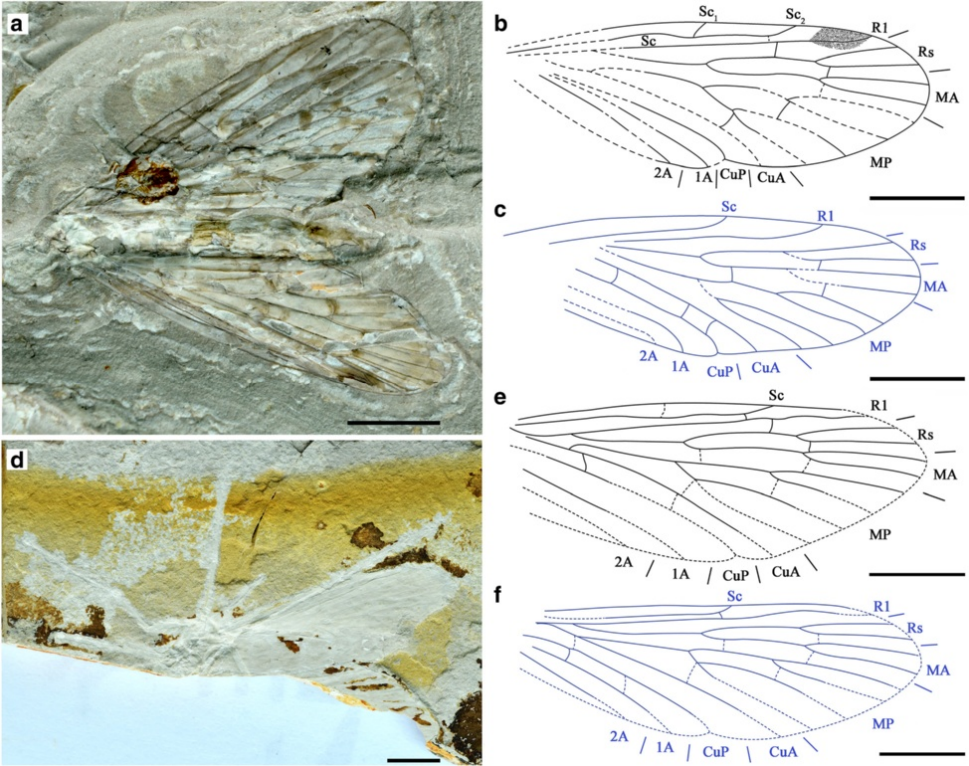
*Lichnomesopsyche prochorista* sp. nov., paratype specimens CNU-MEC-NN-2015001p/c and CNU-MEC-NN-2015016p/c. Specimen CNU-MEC-NN-2015001p: **a** Photograph of part. **b** Overlay drawing of right forewing. **c** Overlay drawing of right hind wing. Specimen CNU-MEC-NN-2015016p: **d** Photograph of part. **e** Overlay drawing of right forewing. **f** Overlay drawing of right hind wing. Scale bars represent 5 mm in (**a**–**f**)

Specimen CNU-MEC-NN-2015001p/c, part and counterpart (Fig. 7a, b, c), with a partially preserved body and well-preserved wings, showing the insect in dorsal view; sex unknown. Forewing length 22.4 mm, width 7.7 mm.

Specimen CNU-MEC-NN-2015016p/c, part and counterpart (Fig. 7d, e, f), an almost complete individual showing the body and one side of the wings; sex unknown. Forewing length 22.0 mm, width 8.3 mm; body length (excluding antennae and proboscis) 24.2 mm; proboscis length at least 10.6 mm; antenna length (as preserved) at least 5.7 mm.

#### Locality and stratigraphic horizon

The specimens were collected near Daohugou Village, Shantou Township, Ningcheng County, in Inner Mongolia of Northeastern China (N41°18.979′, E119°14.318′). The stratigraphic position of the examined material is the Jiulongshan Formation, the late Callovian Stage of the latest Middle Jurassic (164–165 Mya), determined by a ^40^Ar/^39^Ar isotopic age date [49] calibrated to a standard international time scale [49, 50]. The locality and stratigraphic horizon are the same for all other new specimens described herein.

#### Description

Female (based on holotype specimen CNU-MEC-NN-2015002p/c).

Complete insect (Figs. 4 and 5) with well-preserved wings and body.

Head: Triangular in dorsal view, prognathous. Eyes large, widely separated. Proboscis long and slightly curved, apical pseudolabellae absent [3]. Proboscis covered with dense setae or microtrichia but lacking annuli. Antennae distinctly shorter than proboscis, flagellum multiarticulate and filiform, gradually tapering (Fig. 5a, b, e).

Thorax: Pronotum small, meso- and metanotum large, more or less similar to each other. Scutum distinct, but with an indistinct scutellum (Fig. 4c).

Legs: Legs entirely covered with annulated pubescence. The femora of the mid- and hind legs narrow, elongated, with one apical spur. Tibiae long and slender, with one apical spur. Tarsi 5**-**segmented, pretarsus with two claws (Fig. 5f).

Wings: Forewing slender (length/width ratio 3.3:1), apical margin round. Membrane delicate. Sc long, but not extending beyond the MA bifurcation, with only one inclined anterior branch. Pterostigma distinct. Both Rs and MA with two branches. MP bifurcation slightly proximal to the MA + Rs bifurcation, with four long branches. 1A and 2A well developed. Hind wing almost the same as forewing in shape, but slightly smaller and broader (length/width ratio 3.1:1). Sc distinctly short, lacking anterior branches. R1 entering pterostigma, smooth and curved. Pterostigma well defined. The details of the wings are depicted in Fig. 4d.

Abdomen: Female abdomen elongate, tapering apically, with 10 visible segments. Segments 3–7 distinctly long. Segments 8–10 more slender than 2–6, without an enlarged genital bulb (Fig. 5c, d). Cerci at least 2**-**segmented, arising from the segment 10. Basal segments of cerci not fused with each other (Fig. 4c).

Male: (based on paratype specimen CNU-MEC-NN-2015008, Fig. 6).

Head: Triangular in dorsal view. Proboscis straight and siphonate, covered with dense setae. Antennae filiform, shorter than proboscis.

Thorax and Legs: Pronotum small. Meso- and metanotum large, scutum and scutellum indistinct on part and counterpart. A slender right foreleg and parts of left legs are preserved.

Wings: Forewing slightly broader (length/width ratio 3.4:1), with wing venation the same as female. Hind wings almost the same as forewings in shape, but slightly smaller and broader (length/width ratio 3.3:1).

Abdomen: Elongate, with nine visible segments. The first segment closely associated with the metathorax. Segments 2 to 8 normal; nine and ten enlarged, bulbous (Fig. 6c).

*Lichnomesopsyche daohugouensis* Ren, Labandeira and Shih, 2010, p. 723, Fig. 5; p. 730, Plate IV. Holotype: No. CNU-MEC-NN-2005022p/c [21].

#### Revised diagnosis

Hind wing Sc distinctly short; lacking anterior branches. Rs and MA almost forking at the same level. MP forking slightly proximal of the MA+ Rs bifurcation. Thyridium not always evident on both forewings and hind wings.

#### Additional specimens

**Fig. 8:**
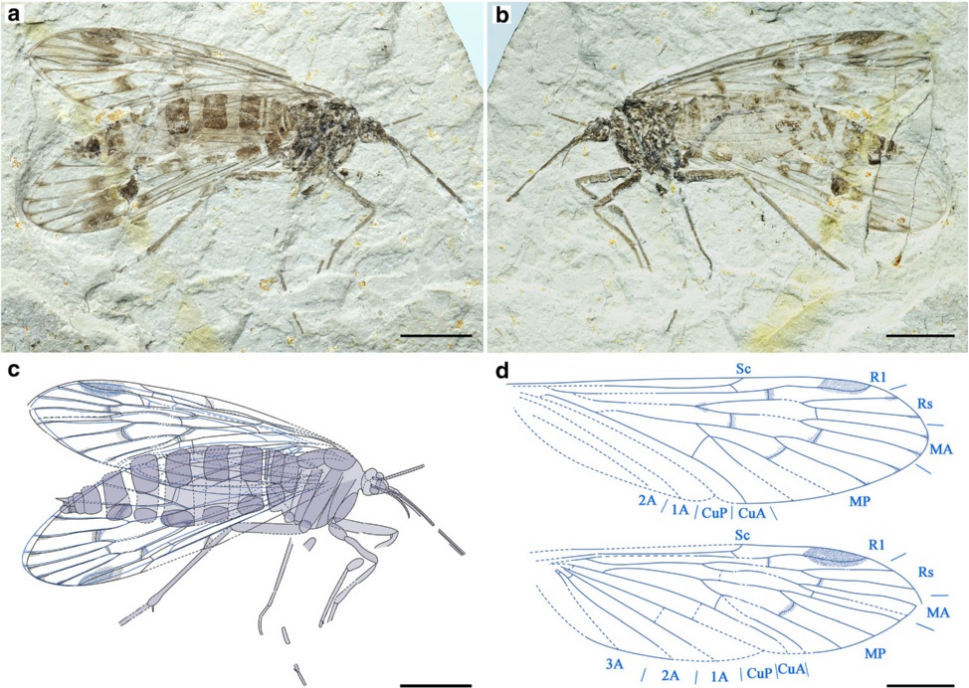
*Lichnomesopsyche daohugouensis* Ren, Labandeira and Shih, 2010, new specimen CNU-MEC-NN-2015003p/c. **a** Photograph of part. **b** Photograph of counterpart. **c** Overlay drawing of part. **d** Overlay drawings of hind wings. Scale bars represent 5 mm in (**a**–**c**), 3 mm in (**d**)

Specimen CNU-MEC-NN-2015003p/c; female, part and counterpart (Fig. 8). Forewing length 21.9 mm, width 7.0 mm; body length (excluding proboscis) at least 23.3 mm; proboscis length at least 6.9 mm.

**Fig. 9:**
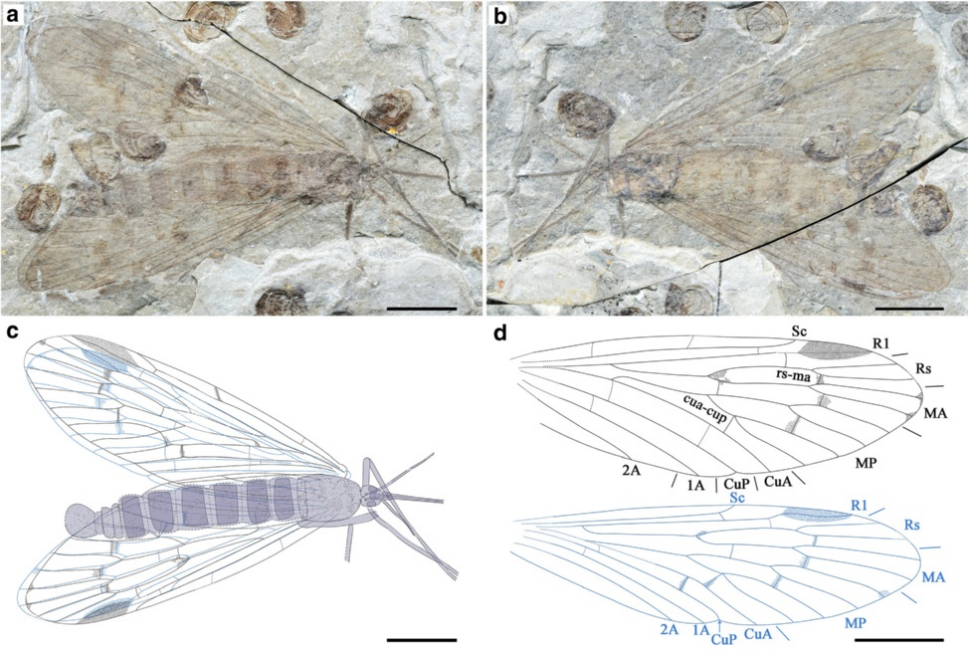
*Lichnomesopsyche daohugouensis* Ren, Labandeira and Shih, 2010, new specimen CNU-MEC-NN-2015011p/c. **a** Photograph of part. **b** Photograph of counterpart. **c** Overlay drawing of part. **d** Overlay drawings of right fore and hind wings. Scale bars represent 5 mm in (**a**–**d**)

Specimen CNU-MEC-NN-2015011p/c; male, part and counterpart (Fig. 9). Forewing length 23.2 mm, width 7.8 mm; body length (excluding proboscis and antennae) at least 22.7 mm; proboscis length at least 4.4 mm.

Specimen CNU-MEC-NN-2015007p/c; female, part and counterpart (Additional file 2: Figure S2). Forewing length 26.4 mm, width 8.3 mm; body length (excluding proboscis) at least 27.4 mm; proboscis length at least 11.3 mm.

Specimen CNU-MEC-NN-2015014; sex unknown (Additional file 3: Figure S3 A, C and D). Forewing length 24.5 mm, width 7.0 mm; body length (as preserved, excluding proboscis) at least 21.4 mm; proboscis length at least 9.0 mm.

Specimen CNU-MEC-NN-2015013; sex unknown (Additional file 3: Figure S3 B). Forewing length 26.8 mm, width 7.7 mm; body length (as preserved, excluding proboscis) at least 25.8 mm; proboscis length at least 10.8 mm.

#### Description

(Based on additional specimens CNU-MEC-NN-2015003p/c, and CNU-MEC-NN-2015011p/c.)

Head: Small and triangular in dorsal view, prognathous. Eyes large, widely separated. Proboscis long and slightly curved at base. Antennae shorter than proboscis, filiform.

Legs: Densely clothed with short setae, never bearing transverse rows of annuli. Coxae and trochanter well-preserved. Femora stout. Tibiae long and slender, with one apical spur. Tarsi 5**-**segmented, pretarsus with two claws.

Wings: Forewing slender (length/width ratio 3.2:1), apical margin rounded. Thyridium not evident. Color pattern well developed around crossveins. Sc long, not extending beyond the MA bifurcation, with only one inclined to anterior branch. Pterostigma distinct. Rs and MA almost forking at the same level, both with two branches. MP bifurcation almost at the same level with MA + Rs bifurcation, MP with four long branches. 1A and 2A well developed. Hind wing almost the same as forewing in size and shape, but broader (length/width ratio 3:1). Sc distinctly short, lacking anterior branches. Pterostigma distinct. Rs and MA both with two branches; MP with 4 long branches. 1A and 2A well developed. The details of the wing venation depicted in Figs. 8d and 9d.

Abdomen: Female (based on additional specimen CNU-MEC-NN-2015003p/c, Fig. 8a–c) abdomen elongate, tapering apically, with 10 visible segments. The first segment fused with metathorax. Segments 3–7 distinctly longer than others. Segments 8–10 more slender than 2–6, without enlarged genital bulb. Cerci at least 2**-**segmented, arising from the segment 10. Basal segments of cerci not fused with each other.

Male (based on additional specimen CNU-MEC-NN-2015011p/c, Fig. 9a–c) abdomen elongate, with nine visible segments; the first one closely associated with the metathorax. The 2–8 segments slender, 9–11 are enlarged, bulbous.

*Vitimopsyche* Novokshonov and Sukatsheva, 2001 [19].

Type species. *Vitimopsyche torta* Novokshonov and Sukatsheva, 2001 [19].

Included Species: Type species, *V. kozlovi* Ren, Labandeira and Shih, 2010 [21] and the new species described herein.

#### Emended diagnosis

In forewing, Sc extends to C beyond the MA bifurcation, some individuals even beyond the Rs bifurcation, with one located anterior. Rs forking considerably more distal of the MA bifurcation, each with two branches. MP originates from the MP + CuA much more proximal of the Rs + MA originating from R1. In the hind wing, Sc short, reaching C not beyond the MA bifurcation. MP originating from the MP + CuA slightly distal or proximal of the Rs + MA from R1.

*Vitimopsyche pristina* sp. nov.

urn: lsid:zoobank.org:act:9942EAC8-55CB-4D8C-90AB-0127559BA8FC

#### Etymology

The specific name *pristina* originates from the Latin, *pristinus*, meaning “primordial”, which refers to the geological time interval of occurrence of the new species is earlier than other species in this genus.

#### Diagnosis

The new species resembles *V. torta* and *V. kozlovi* in venational features, but is distinguished from them by the Sc reaching to the C beyond the Rs bifurcation in the forewing; and the hind wing MP originating from stem of the MP + CuA distal of Rs + MA from R1. It also differs from *V. torta* by the Sc with only one anterior branch in forewing.

#### Holotype

Specimen CNU-MEC-NN-2015009 of one individual with partly preserved body and wings; female. Forewing length 18.7 mm, width 6.2 mm; proboscis length (as preserved) is 4.5 mm.

#### Description

**Fig. 10:**
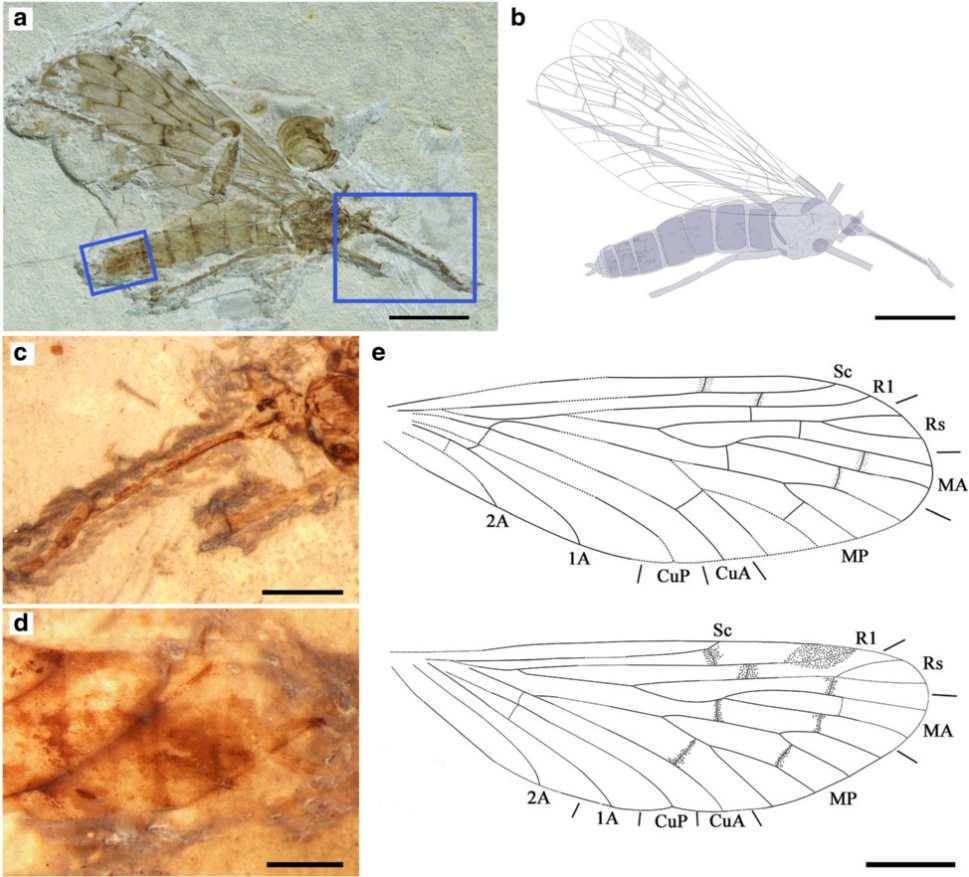
*Vitimopsyche pristina* sp. nov., holotype CNU-MEC-NN-2015009. **a** Photograph of specimen CNU-MEC-NN-2015009. **b** Overlay drawing of CNU-MEC-NN-2015009. **c** Photograph under ethanol of head and proboscis. **d** Photograph under ethanol of female genitalia. **e** Overlay drawing of left fore and hind wings. Scale bars represent 5 mm in (**a**) and (**b**), 2 mm in (**c**), 1 mm in (**d**), and 3 mm in (**e**)

The specimen has only one side of the wings preserved (Fig. 10a, b), while the fore- and hind wings are overlapping.

Head: Triangular in dorsal view, prognathous. Antennae unknown; eyes not well preserved. Frons and clypeus not noticeable. Proboscis incomplete; lacking fine annuli of microtrichia, pseudolabellae not present (Fig. 10c).

Thorax: Not discernible.

Legs: Slender, incompletely preserved.

Wings: Forewing broadened (length/width ratio: 3.0). Membrane delicate. Sc long, reaching C beyond Rs bifurcation, not forking terminally, with only one anterior branch. R1 single, slightly curved near its ending. Pterostigma not well preserved. Both Rs and MA with two branches; MA stem and MA_1_ branch forming a distinct S-shape; one cross-vein between MA_1_ and MA_2_. MP with four long branches, MP_1+2_ forking distal to MP_3+4_ bifurcation. Thyridium not evident. CuA and CuP single; 1A and 2A single and well developed; one crossvein between CuP and 1A, and one between 1A and 2A.

Hind wing almost the same as forewing in shape but smaller. Right hind wing length 19.5 mm, width 6.2 mm; length/width ratio 3.2. Sc short, forking and reaching C at about the same level of the MA bifurcation, lacking anterior branches. Pterostigma well defined. MP originating from the stem of MP + CuA distal of the Rs + MA originating from R1 (Fig. 10e).

Abdomen: Elongate, tapering apically, with 10 visible segments. The first segment fused with metathorax, Segments 8–10 more slender than segments 2–6, without an enlarged genital bulb (Fig. 10d).

### Results of phylogenetic analysis

**Fig. 11:**
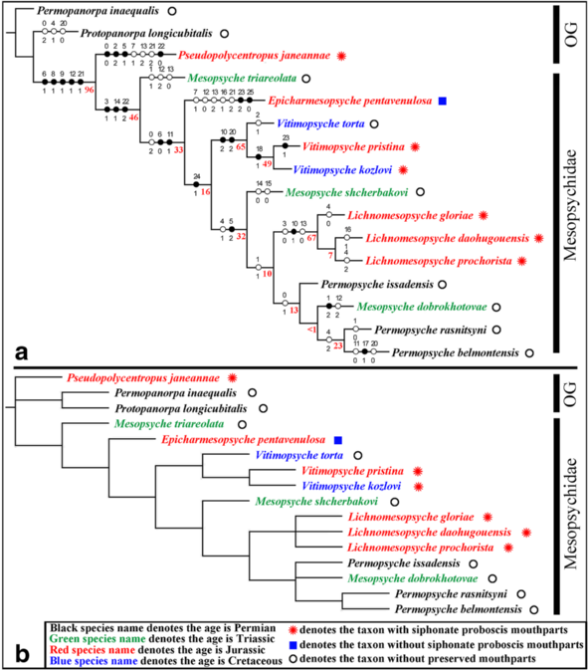
Results of phylogenetic analyses (NONA and PAUP). **a** The most parsimonious tree with bootstrap support by NONA. **b** The consensus tree by PAUP. White circles indicate homoplasious characters, and the black circles indicate non-homoplasious characters. The numbers above branches are character numbers, below branches are character states, and the red numbers below the branches are bootstrap values in (**a**)

The maximum parsimony analysis by NONA yielded only one most parsimonious tree (Fig. 11a; tree length = 70 steps, consistency index (CI) = 0.60, retention index (RI) = 0.68). Morphological characters were optimized with parsimony on the most parsimonious tree, showing only unambiguous changes. The bootstrap value results are shown in Fig. 11a.

The analysis by PAUP produced two most parsimonious trees (Additional file 4: Figure S4), and the consensus tree is shown in Fig. 11b (tree length = 73 steps, CI = 0.5735, RI = 0.6630). Two characters are uninformative (characters 23 and 25), but we did not exclude them in our analyses because they provide additional information for individual tree branches. There are 24 parsimony-informative characters.

The monophyly of the Mesopsychidae is supported by three synapomorphic characters (Fig. 11a). They are: 1), the forewing Rs + MA vein forks from the R1 vein far distal to the MP vein that originates from CuA (Character 3:1); 2), the forewing CuP vein occurs almost at the same level or distal to mid-length of the wing (Character 14:1); and 3), the anal area is expanded on the forewing (Character 22:2). One of the basalmost taxa of the Mesopsychidae is *Epicharmesopsyche*, whose sister-group relationship with other species of the ingroups is supported by one synapomorphic character, namely that the antennae is short and filiform (Character 24:1). The basal position of *Epicharmesopsyche* is indirectly determined, as this genus possesses one described species and is monotypic.

The monophyly of *Vitimopsyche* is supported by two synapomorphic characters. The first feature is the forewing MP forking distal to the Rs + MA bifurcation (Character 10:2). The second character is the forewing crossvein mp2-mp3 occurring considerably distal to the MP1 + 2 bifurcation point (Character 20:2). The systematic position of the new species, *Vitimopsyche pristina* sp. nov., also is confirmed by the one synapomorphic character of the hind wing Sc vein of medium length that is present slightly proximal to the MA vein bifurcation (Character 23:1).

The monophyly of *Lichnomesopsyche* is supported by one unambiguous character: the forking of the MP and Rs + MA veins very close to or at the same level in the forewing (Character 10:1). In addition, there are two non-homologous characters of: 1) a Rs + MA bifurcation from the R1 vein present slightly distal to where the MP vein originates from the CuA vein (Character 3:0); and 2) the forewing cup-cua crossvein is far distal to the MP vein and originates from the CuA vein (Character 13:0). These features render the three species of *Lichnomesopsyche,* including *Lichnomesopsyche prochorista* sp. nov., as forming one clade equivalent to a genus.

*Mesopsyche*, in contrast, is a paraphyletic group, as the three species are scattered across the phylogenetic tree. The sister group of *Mesopsyche triareolata* is supported by two homologous characters: 1) absence of a crossvein between R1 and C (Character 6:0); and 2) the posterior margin of the forewings bearing an emargination at the CuP vein apex (Character 11:1). The basal position of *Mesopsyche triareolata* clade, however, is only indirectly determined. The clade of *Mesopsyche shcherbakovi* and its sister group is supported by one synapomorphic character, the MP1 + 2 forking is proximal to the MA forking in the forewing (Character 5:2). We suggest revising the taxonomy of these two species, pending future discovery and study of additional new specimens.

*Mesopsyche dobrokhotovae* exhibits close affinity to species of *Permopsyche*, particularly in that the base of the CuA vein is oblique to transverse and not inclined backwards [21]. *Mesopsyche dobrokhotovae* mainly conforms to the character of *Permopsyche*. The systematic position of *Mesopsyche dobrokhotovae* should be revised, a conclusion confirmed by one synapomorphic character, the forewing Sc fore-branch (Sc_1_) vein ending at a point proximal to the 1A vein terminus (Character 1:2). Consequently, we propose to transfer *Mesopsyche dobrokhotovae* to the genus *Permopsyche*, as *Permopsyche dobrokhotovae* (Novokshonov, 1997) comb. nov. Three species of *Permopsyche* and *Mesopsyche dobrokhotovae* are shown as a sister clade to *Lichnomesopsyche.* It is interesting to note the geochronologically wide age distinctions among these species, ranging from the Late Permian to Middle Jurassic.

The resulting consensus tree by PAUP (Fig. 11b) is very similar to the tree by NONA (Fig. 11a). Only two differences are present. One distinction is the relationship among the three outgroups. The results from NONA exhibits a closer relationship of *Pseudopolycentropus janeannae* and the ingroups, whereas the result by PAUP shows a parallel relationship of three outgroups and an ingroup. The other feature resolved by NONA is the interspecies relationship of the two genera, *Lichnomesopsyche* and *Permopsyche*, which was not resolved by PAUP.

Based on the two phylogenetic analyses, Mesopsychidae is a monophyletic group; in addition, two of its genera, *Vitimopsyche* and *Lichnomesopsyche*, also are monophyletic (Fig. 11). *Permopsyche* and *Mesopsyche* are paraphyletic. *Epicharmesopsyche* is a monotypic species. Although the relationships among the genera are incompletely resolved, we have carried out a geometric morphometric analyses to supplement the results of our phylogenetic analyses.

### Results of geometric morphometric analysis of forewings

**Fig. 12:**
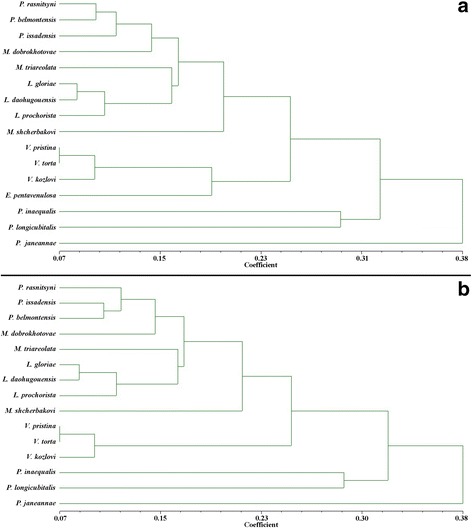
Resulting Trees for the geometric morphometric analyses. **a** Tree 1 for 38 landmarks (including 3 outgroups, no crossvein characters). **b** Tree 2 for 42 landmarks (including 3 outgroups, two crossvein characters)

For the first study, a forewing geometric analysis consisted of 13 ingroups, three outgroups, 38 landmarks, but no crossvein characters. An unweighted pair-group method that employs arithmetic averages (UPGMA) produced Tree 1 (Fig. 12a). The results of this study show the phenetic relationships without consideration of evolutionary relationships among the sixteen scorpionfly wing samples. A second study consisted of twelve ingroups and three outgroups, using 42 landmarks and two crossvein characters that produced Tree 2 (Fig. 12b).

From the resulting geometric morphometric Trees 1 and 2 (Fig. 12a, b), which are nearly congruent, we obtain the following five conclusions. First, *Lichnomesopsyche prochorista* sp. nov. has wing venation most similar to those of the other two species of the genus *Lichnomesopsyche*. Second, *Vitimopsyche pristina* sp. nov. has wing venation most similar to those of the two other species of the genus *Vitimopsyche*. Third, the group containing three species of *Permopsyche* and *M. dobrokhotovae* has very similar wing venation, which is different from that of the genus *Lichnomesopsyche* and other taxa in Mesopsychidae. Fourth, the venation of *M. triareolata* and *M. shcherbakovi* are unique and distinct from other taxa or groups of Mesopsychidae. Fifth, *Epicharmesopsyche pentavenulosa* is a unique taxon with venation different from all other ingroups of Mesopsychidae, shown in Tree 1 (Fig. 12a).

The result of the geometric morphometric analyses also indicates that *Mesopsyche* is not monophyletic, and that *Mesopsyche dobrokhotovae* has an affinity with species of *Permopsyche*. This is consistent with the results of our phylogenetic analyses and with comparisons of the original description, line drawings, and photographs. As a result, we further validate our proposal for transferal of *Mesopsyche dobrokhotovae* to *Permopsyche dobrokhotovae* (Novokshonov, 1997) comb. nov. [18], and consider that the taxonomic positions of the other two species should be revised, pending availability of new specimens and additional study. Based on current information, it is hypothesized that the geochronologic longevity and geographic distribution of *Permopsyche* extended from the Upper Permian of Russia and Australia to the Upper Triassic of the Ukraine [14].

## Discussion

### Taxonomy and phylogeny of mesopsychidae

Although most of the examined specimens have preserved wings rich in structural characters, due to limited number of fossil specimens described, this study is the first attempt to compile data and conduct phylogenetic and geometric morphometric analyses based on previously described and new species. These phylogenetic results support the hypothesis that Mesopsychidae is a monophyletic group. The systematic positions of the two newly described species, *L. prochorista* sp. nov. and *V. pristina* sp. nov. are validated. *Vitimopsyche pristina* sp. nov. represents the earliest fossil record of the genus *Vitimopsyche*, thus, extending the genus from the Lower Cretaceous to the latest Middle Jurassic. Based on our phylogenetic and geometric morphometric analyses, we propose erection of the taxon *Permopsyche dobrokhotovae* (Novokshonov, 1997) comb. nov., and suggest a taxonomic revision of the two other species of *Mesopsyche* in the future.

### Comparison of phylogenetic and geometric morphometric analyses

For a better perspective regarding relationships among representative genera and species of Mesopsychidae, we applied two methods for analyses of the same specimens. Both approaches were useful for understanding the intergeneric relationships among taxa and to infer the systematic positions of the two new species. In the phylogenetic analysis, many morphological characteristics were chosen, including abundant characters of the forewing, and a few characters of the hind wing and body. By comparison, the geometric morphometric analyses only employed forewing landmarks. The character states of phylogenetic analyses are discrete states, but landmark analysis used in geometric morphometric analyses allow for continuous changes of a particular landmark character. Because many landmarks occur on the wing margin, the geometric morphometric analyses include comparisons of wing shapes [28], which is not part of the phylogenetic analysis. Alternatively, character states of phylogenetic analyses allow some missing character data, which frequently occur for fossil specimens. The geometric morphometric analyses do not allow for missing landmarks, unless they are added by invoking assumptions. Consequently, both methodologies, based on the same set of specimens, complement each other to provide a better understanding of the phylogenetic and phenetic relationships among these specimens.

The resulting trees from the two methods exhibit a fundamental consistency and similarity, but, with two obvious differences as shown in Additional file 5: Figure S5: The first distinction is the varying placement of *Mesopsyche triareolata.* In the tree shown in Additional file 5: Figure S5B, *Mesopsyche triareolata* and the species of *Lichnomesopsyche* are in one clade, whereas in the phylogenetic tree in Additional file 5: Figure S5A, *M. triareolata* and the rest of ingroup species are sister groups. The second distinction is that the sister groups of *Epicharmesopsyche pentavenulosa* are different. As mentioned previously, the most basal position of *Mesopsyche triareolata* is indirectly supported by its sister group, which are represented by five landmarks: 2 vs. 35 and 37, 38 vs. 19. Since geometric morphometric analyses treat all landmarks equally and without weighting, the positions of other landmarks, such as 33 landmarks for Tree 1 (Fig. 12a) and 37 for Tree 2 (Fig. 12b), seem to have more of a significant impact on relative venational similarity and the phenetic placement of *M. triareolata* in Trees 1 and 2.

### Origin and early evolution of the mesopsychid siphonate proboscis

The phylogenetic analysis of Mesopsychidae sheds light on the origin of the siphonate proboscis and in its closest related family, the Pseudopolycentropodidae. The second most basal Mesopsychidae, *Epicharmesopsyche pentavenulosa,* apparently lacked a proboscis [23], although the mouthpart condition of the two outgroups, *Permopanorpa inaequalis*, *Protopanorpa longicubitalis* and *Mesopsyche triareolata,* the most basal mesopsychid, remain unknown. Additionally, the proboscis of Pseudopolycentropodidae is structurally very different from that of the Mesopsychidae [8, 12]. Consequently, it appears that the proboscis was evolved independently in the Pseudopolycentropodidae, also in the *Vitimopsyche* and possibly in the *Lichnomesopsyche* clades of the Mesopsychidae. Nevertheless, the mouthpart conditions of the intervening *Mesopsyche shcherbakovi* and the species of *Permopsyche,* the sister clade to *Lichnomesopsyche,* remain unknown. More broadly, within late Paleozoic to mid Mesozoic Mecoptera, the Nedubroviidae [9] and Aneuretopsychidae [3, 10] also have long-proboscid, siphonate mouthparts. This suggests that the long-proboscid condition might independently have originated four or possibly five (assuming independent originations in *Vitimopsyche* and *Lichnomesopsyche*) times within early Mecoptera.

The pattern of the origin of the siphonate proboscis four or five times in Mecoptera contrasts dramatically with that of Lepidoptera in which evidence indicates a single origination event for a considerably more diverse clade [51]. Another significant difference is that the lepidopteran proboscis is derived from paired maxillary galeae [52], whereas the mecopteran proboscis consists of paired labial elements. Unlike lepidopterans, the construction of the mecopteran’s labially derived proboscis is based on elongation of paired labial elements such as glossae or palps that are conjoined to anatomically form the siphon for the imbibition of fluids [3]. The origin of the mecopteran proboscis likely has an evolutionary developmental explanation. Smith and Jockusch [53] recently have documented such a transformation in embryonic mouthpart development in the beetle, *Tribolium castaneum*, which has well developed, chewing, mandibulate mouthparts. In *T. castaneum* the specificity functions of the genes *extradenticle* (*ext*) and *homothorax* (*hth*) were subject to gene knockdown, providing interference RNA (RNAi) phenotypes. The resulting adult mouthpart malformations of this mandibulate insect included modifications of the maxillary and labial regions. The maxillary region displayed enlargement of some elements, transformation of proximal structures to more distal identity, and inter-element fusions, though not formation of the anatomical precursor to siphonate mouthparts.

**Fig. 13:**
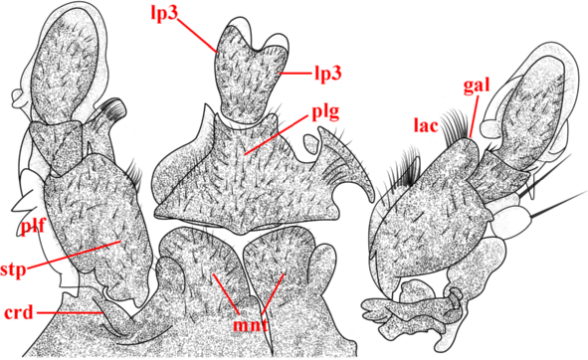
An adult mouthpart phenotype with medial fusion of labial palps in *Tribolium castaneum* resulting from larval RNAi knockdown of the genes *homothorax* (*hth*) and *extradenticle* (e*xt*). The example represents a more severe case in the transformation of the loss of labial glossa and paraglossal elements, significant size reduction of the mentum (mnt) and prementum, and fusion of the labial palps into a single medial structure. Abbreviations for maxillary elements are: crd, cardo; stp, stipes; plf, palpiger; lac, lacinia; and gal, galea. Abbreviations for labium are: plg, paliger, lp3 labial palp segment 3. This figure was redrawn from Fig. 1P of Smith and Jockusch (2014) [53]

By contrast, the adult labium in *T. castaneum* underwent a major structural transformation that could represent the initial stages of a siphonate proboscis (Fig. 13). Upon knockdown of the *ext* and *hth* genes by RNAi treatment, there was: 1) a change of more proximal element regions to a more distal identity, 2) a narrowing of the prementum and mentum basal regions; 3) elongation of the palpiger sclerite that bears the labial palps, and most significantly, 4) deletion of the ligula and most of the prementum occurring between the labial palps, and 5) reduction and medial fusion of the labial palps into a single, somewhat prolonged structure [53]. Other knockdown genes, such as *proboscipedia* (*pb*), instead resulted in transformation of the labial palpi into a leg-like structures, indicating that it is the *ext* and *hth* genes in *T. castaneum* that largely is responsible for alteration of nominal mandibulate mouthparts into an incipiently siphonate condition. It is unclear if the medially conjoined labial structure was tubular in nature, or if it had any connection to an anatomical mouth. Repeated rounds of suppression of *ext* and *hth* genes could explain initial stages of the proboscis origin among early Mecoptera.

## Conclusions

Phylogenetic results support that Mesopsychidae is a monophyletic group. The systematic positions of *L. prochorista* sp. nov. and *V. pristina* sp. nov. are confirmed as belonging, respectively, to the *Lichnomesopsyche* and *Vitimopsyche* lineages. The existence of *Vitimopsyche pristina* sp. nov. extends this genus from the Lower Cretaceous to the latest Middle Jurassic. Based on our phylogenetic analyses and geometric morphometric analyses, we propose to transfer *Mesopsyche dobrokhotovae* to *Permopsyche*, and suggest revising the taxonomy of the other two species, pending the discovery and study of new specimens. In addition, tree topology supports the origin of the siphonate proboscis in the common ancestor to the *Vitimopsyche* and *Lichnomesopsyche* clades, possibly in an independent fashion, and siphon loss in *Epicharmesopsyche*. It is suggested that the long-proboscid condition may have independently originated up to five times within early Mecoptera, namely Nedubroviidae, Aneuretopsychidae, Pseudopolycentropodidae and Mesopsychidae, with possible separate originations in *Vitimopsyche* and *Lichnomesopsyche*. It is hypothesized that repeated rounds of suppression of *ext* and *hth* genes could explain initial stages of the proboscis origin among early Mecoptera. Future phylogenetic and geometric morphometric studies, and new mouthpart examinations of additional fossil specimens and better preserved material will augment understanding of the origin, evolution and phylogeny of Mesopsychidae and their siphonate proboscides.

## Additional files

Additional file 1: Figure S1. The selection of the morphological characters in forewing. (A) *Lichnomesopsyche daohugouensis*, from Ren, Labandeira, and Shih, 2010 [21], represent the ingroup species. (B) *Protopanorpa longicubitalis*, from Bashkuev, 2010 [39]. (C) *Pseudopolycentropus janeannae*, from Ren et al., 2010 [8]. The veins in brown denote the character 2, in green denote the character 6, and in purple denote the character 7. The red numbers denote the number of branches for characters 8 and 9, the blue numbers show the positions and number-counting of crossveins for character 21. Green lines denote the middle of the wings, character 14. The areas shown in blue denote the anal area in the forewing, character 22. Green circles show the spots, character 16. Blue angles denote the inclination of the cup-cua, character 12. Red dots denote other forewing characters representing the relative positions of the bifurcating points and intersections of veins and wing margin. (TIF 1300 kb) Additional file 2: Figure S2.
*Lichnomesopsyche daohugouensis* Ren, Labandeira and Shih, 2010, new specimen CNU-MEC-NN-2015007p/c. (A) Photograph of part. (B) Photograph of counterpart. (C) Overlay drawing of part. (D) Line drawings of hind wings. Scale bars represent 5 mm in (A)–(D). (TIF 5102 kb) Additional file 3: Figure S3. Photographs of *Lichnomesopsyche daohugouensis* Ren, Labandeira and Shih, 2010, new specimens CNU-MEC-NN-2015013 and CNU-MEC-NN-2015014. (A) Specimen CNU-MEC-NN-2015014. (B) Specimen CNU-MEC-NN-2015013. (C) Female genitalia of specimen CNU-MEC-NN-2015014 under ethanol. (D) Head and part of the forelegs of specimen CNU-MEC-NN-2015014 under ethanol. Scale bars represent 5 mm in (A) and (B), 2 mm in (C) and (D). (TIF 6972 kb) Additional file 4: Figure S4. Results of phylogenetic analysis by PAUP. (A) The most parsimonious tree 1. (B) The most parsimonious tree 2. (TIF 295 kb) Additional file 5: Figure S5. Comparison of the most parsimonious tree and the Tree 1 of geometric morphometric analyses. (A) The most parsimonious tree (without showing the characters and their states, by NONA). (B) Tree 1 of geometric morphometric analyses (Tree 1 flipped to make it easier to compare with the phylogenetic tree, and without showing the tree length). The red rectangles denote the different positions of *Mesopsyche triareolata*. The green rectangles denote the different positons of *Epicharmesopsyche pentavenulosa*. (TIF 477 kb)
