# Supplementary figures and images for: New data from the Middle Jurassic of China shed light on the phylogeny and origin of the proboscis in the Mesopsychidae (Insecta: Mecoptera)
Source: BMC Evol Biol. 2016 Jan 4;16:1. doi: 10.1186/s12862-015-0575-y (PMC4700641; doi:10.1186/s12862-015-0575-y)

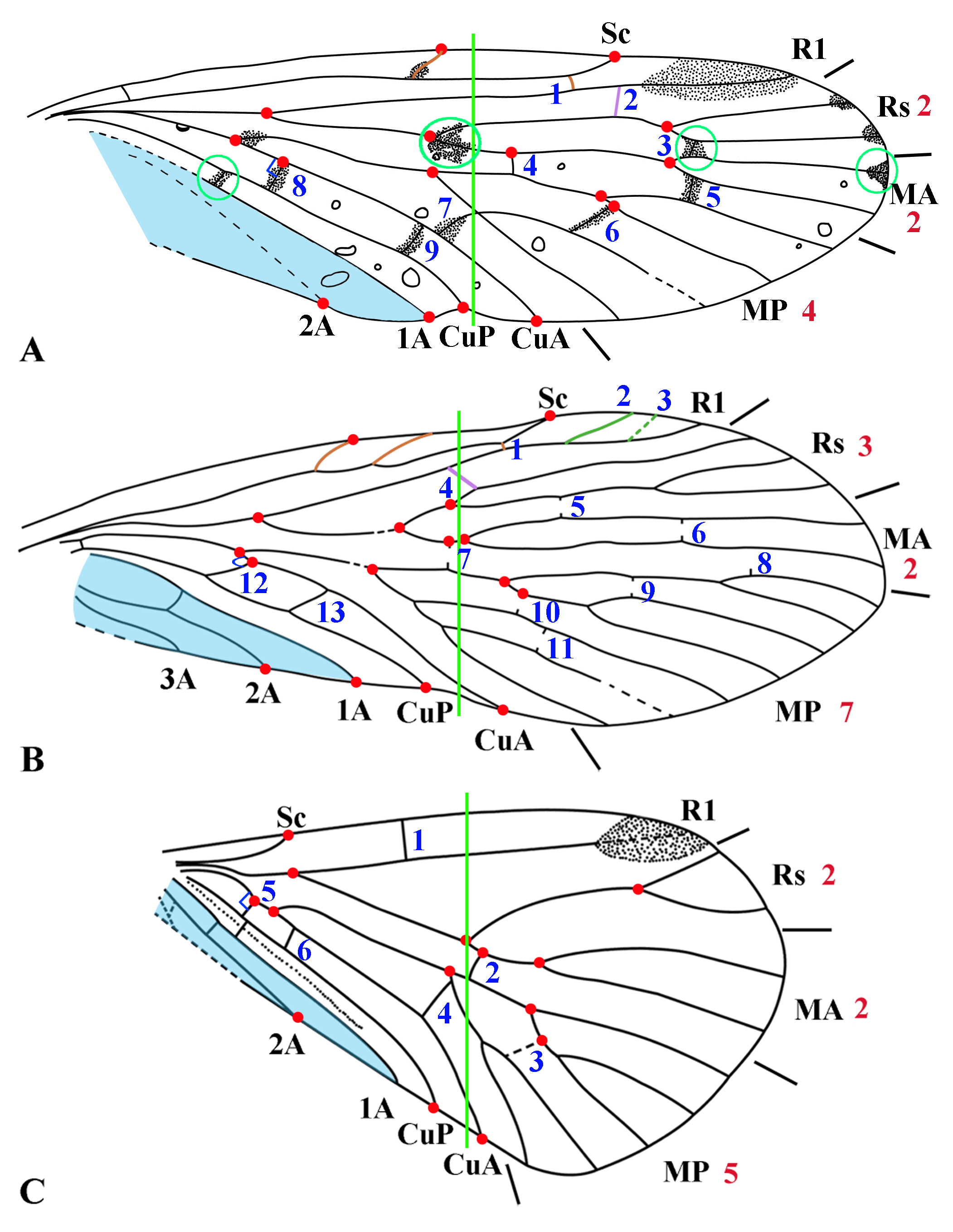

Supplement: Additional file 1: Figure S1. — The selection of the morphological characters in forewing. (A) Lichnomesopsyche daohugouensis, from Ren, Labandeira, and Shih, 2010 [21], represent the ingroup species. (B) Protopanorpa longicubitalis, from Bashkuev, 2010 [39]. (C) Pseudopolycentropus janeannae, from Ren et al., 2010 [8]. The veins in brown denote the character 2, in green denote the character 6, and in purple denote the character 7. The red numbers denote the number of branches for characters 8 and 9, the blue numbers show the positions and number-counting of crossveins for character 21. Green lines denote the middle of the wings, character 14. The areas shown in blue denote the anal area in the forewing, character 22. Green circles show the spots, character 16. Blue angles denote the inclination of the cup-cua, character 12. Red dots denote other forewing characters representing the relative positions of the bifurcating points and intersections of veins and wing margin. (TIF 1300 kb) [file 12862_2015_575_MOESM1_ESM.tif]

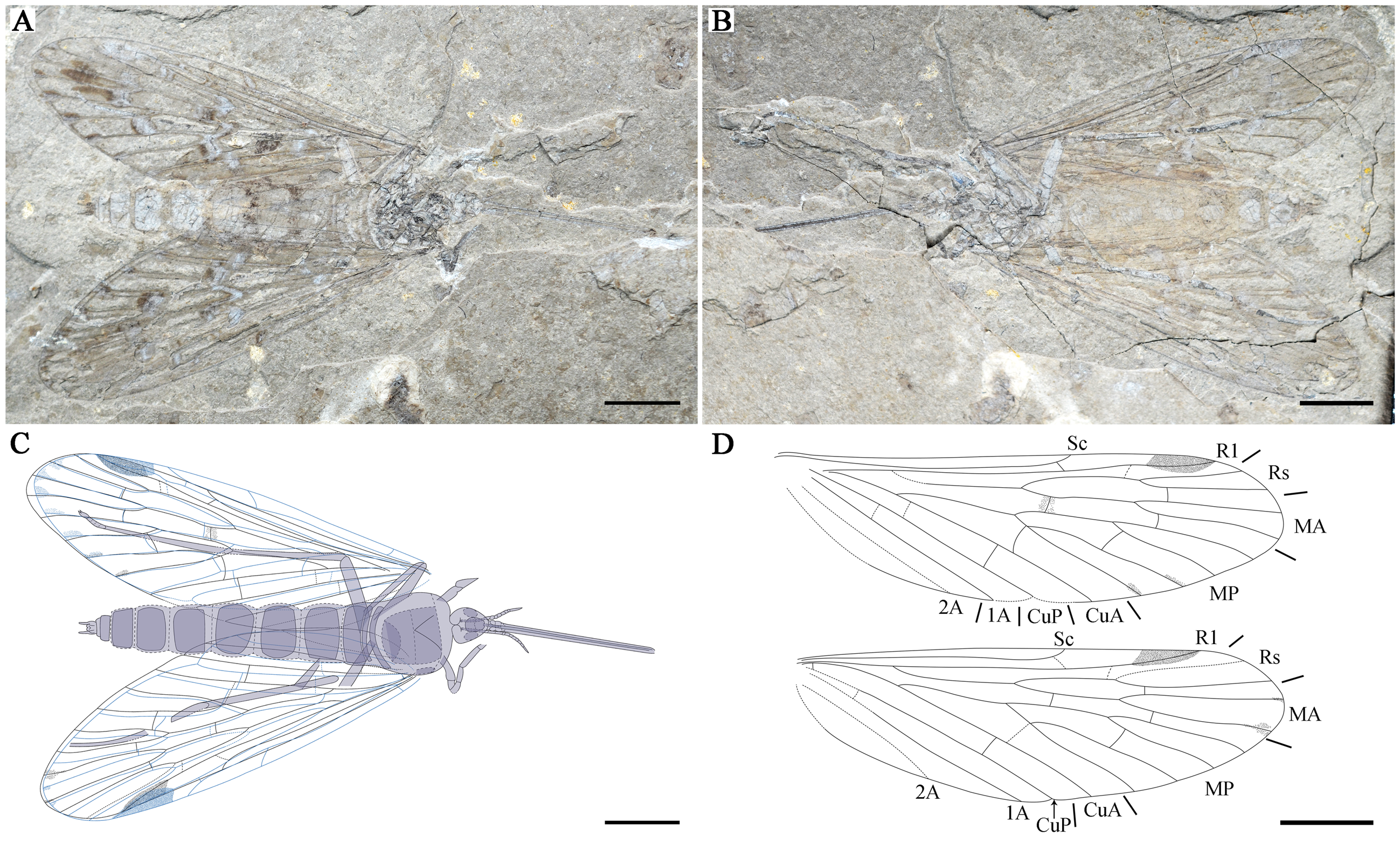

Supplement: Additional file 2: Figure S2. — Lichnomesopsyche daohugouensis Ren, Labandeira and Shih, 2010, new specimen CNU-MEC-NN-2015007p/c. (A) Photograph of part. (B) Photograph of counterpart. (C) Overlay drawing of part. (D) Line drawings of hind wings. Scale bars represent 5 mm in (A)–(D). (TIF 5102 kb) [file 12862_2015_575_MOESM2_ESM.tif]

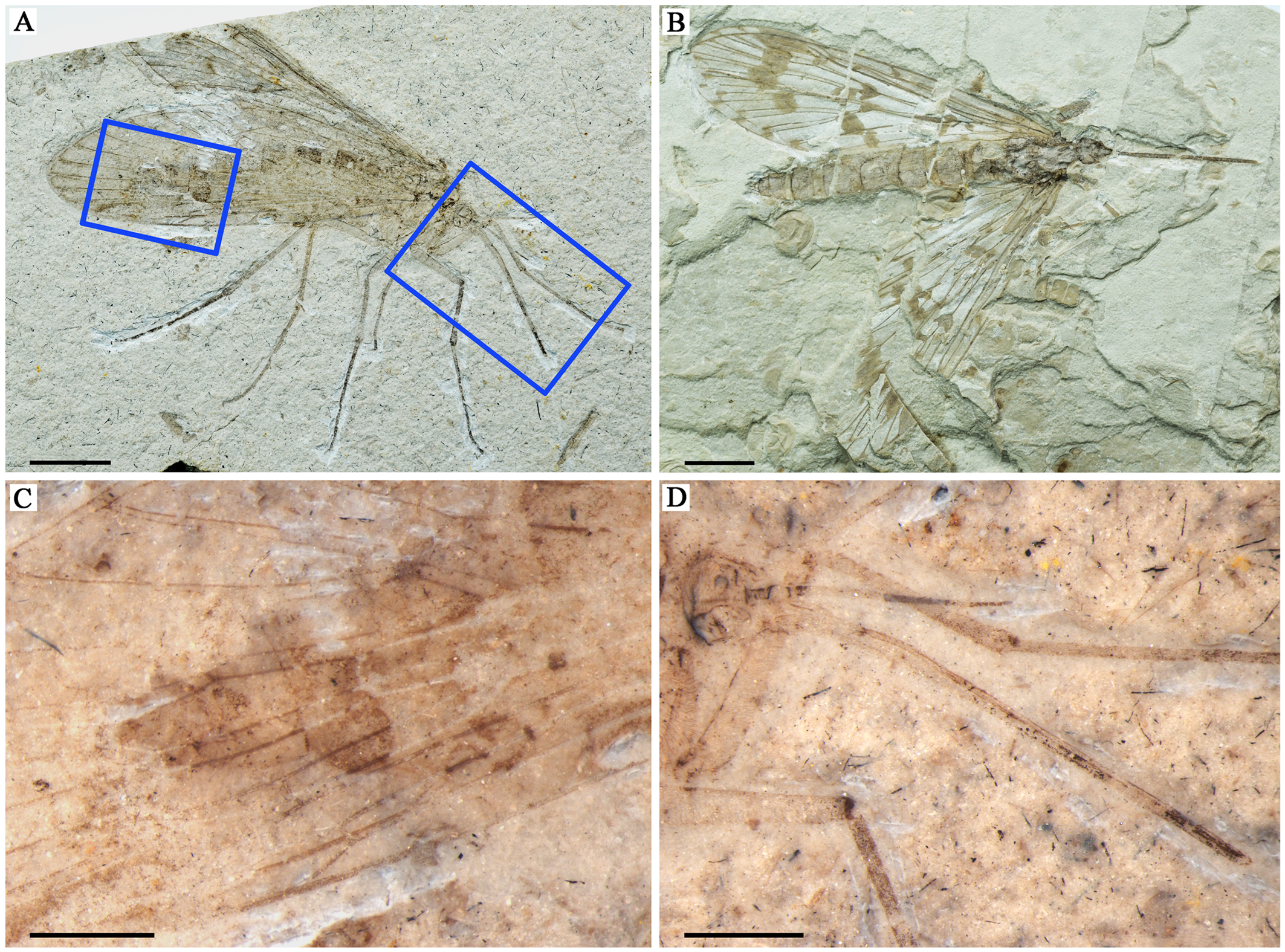

Supplement: Additional file 3: Figure S3. — Photographs of Lichnomesopsyche daohugouensis Ren, Labandeira and Shih, 2010, new specimens CNU-MEC-NN-2015013 and CNU-MEC-NN-2015014. (A) Specimen CNU-MEC-NN-2015014. (B) Specimen CNU-MEC-NN-2015013. (C) Female genitalia of specimen CNU-MEC-NN-2015014 under ethanol. (D) Head and part of the forelegs of specimen CNU-MEC-NN-2015014 under ethanol. Scale bars represent 5 mm in (A) and (B), 2 mm in (C) and (D). (TIF 6972 kb) [file 12862_2015_575_MOESM3_ESM.tif]

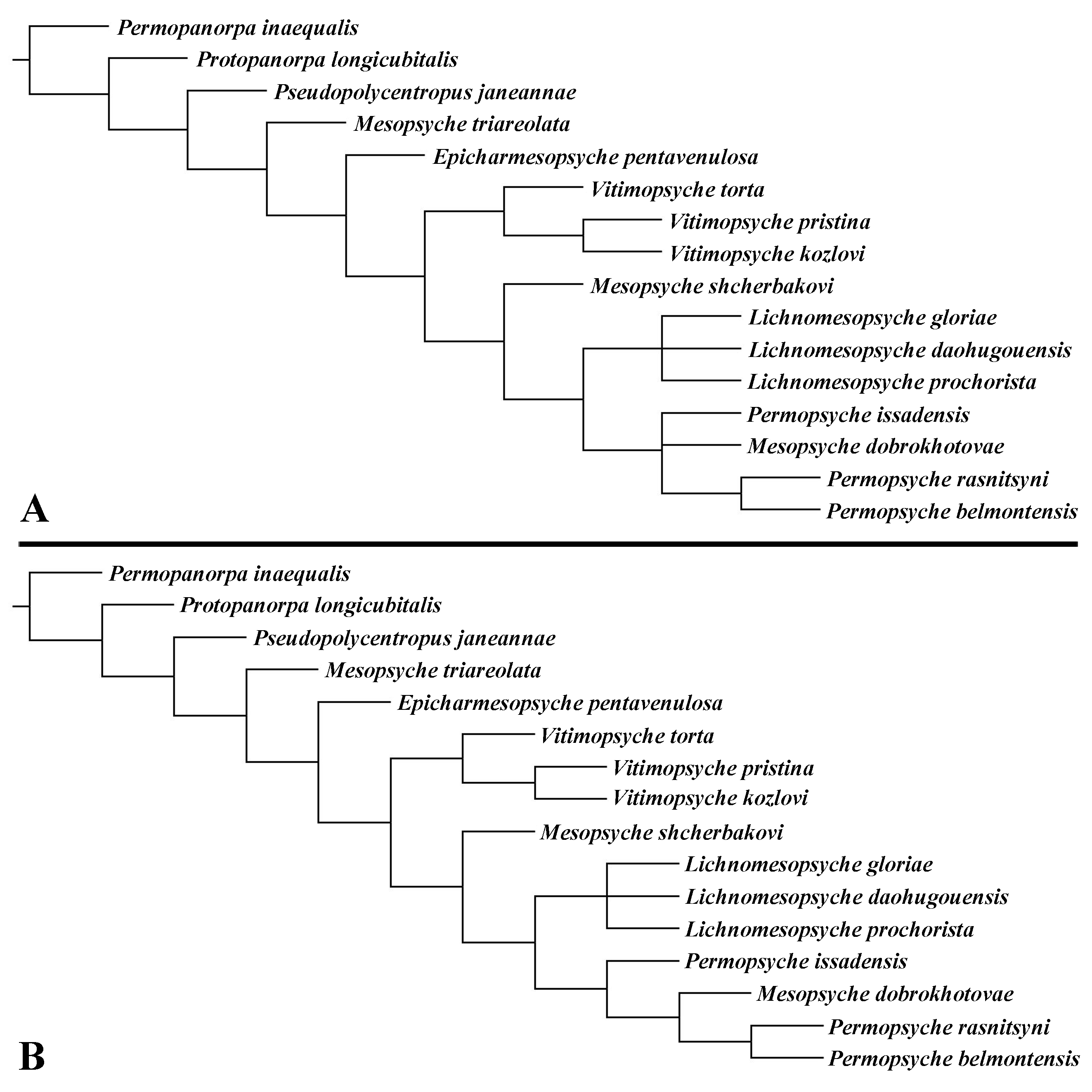

Supplement: Additional file 4: Figure S4. — Results of phylogenetic analysis by PAUP. (A) The most parsimonious tree 1. (B) The most parsimonious tree 2. (TIF 295 kb) [file 12862_2015_575_MOESM4_ESM.tif]

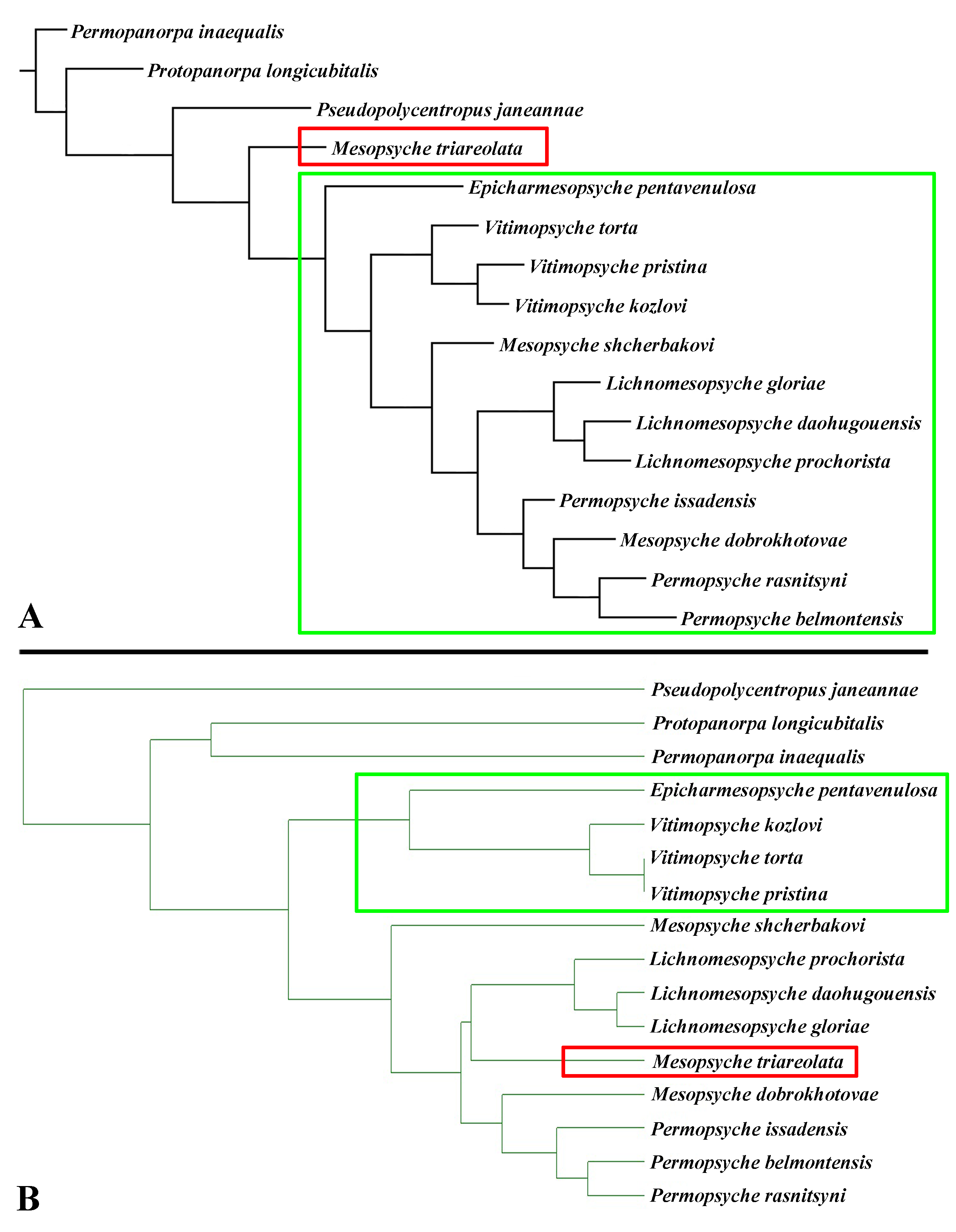

Supplement: Additional file 5: Figure S5. — Comparison of the most parsimonious tree and the Tree 1 of geometric morphometric analyses. (A) The most parsimonious tree (without showing the characters and their states, by NONA). (B) Tree 1 of geometric morphometric analyses (Tree 1 flipped to make it easier to compare with the phylogenetic tree, and without showing the tree length). The red rectangles denote the different positions of Mesopsyche triareolata. The green rectangles denote the different positons of Epicharmesopsyche pentavenulosa. (TIF 477 kb) [file 12862_2015_575_MOESM5_ESM.tif]
